# mRNA vaccines in tumor targeted therapy: mechanism, clinical application, and development trends

**DOI:** 10.1186/s40364-024-00644-3

**Published:** 2024-08-31

**Authors:** Yu Gao, Liang Yang, Zhenning Li, Xueqiang Peng, Hangyu Li

**Affiliations:** 1 Department of General Surgery, The Fourth Affiliated Hospital, China Medical University, Shenyang, 110032 China; 2 Department of Oromaxillofacial-Head and Neck Surgery, School and Hospital of Stomatology, China Medical University, Liaoning Province Key Laboratory of Oral Disease, Shenyang, 110001 China

**Keywords:** mRNA vaccines, Tumor-targeted therapy, Mechanism, Clinical application, Development trends

## Abstract

Malignant tumors remain a primary cause of human mortality. Among the various treatment modalities for neoplasms, tumor vaccines have consistently shown efficacy and promising potential. These vaccines offer advantages such as specificity, safety, and tolerability, with mRNA vaccines representing promising platforms. By introducing exogenous mRNAs encoding antigens into somatic cells and subsequently synthesizing antigens through gene expression systems, mRNA vaccines can effectively induce immune responses. Katalin Karikó and Drew Weissman were awarded the 2023 Nobel Prize in Physiology or Medicine for their great contributions to mRNA vaccine research. Compared with traditional tumor vaccines, mRNA vaccines have several advantages, including rapid preparation, reduced contamination, nonintegrability, and high biodegradability. Tumor-targeted therapy is an innovative treatment modality that enables precise targeting of tumor cells, minimizes damage to normal tissues, is safe at high doses, and demonstrates great efficacy. Currently, targeted therapy has become an important treatment option for malignant tumors. The application of mRNA vaccines in tumor-targeted therapy is expanding, with numerous clinical trials underway. We systematically outline the targeted delivery mechanism of mRNA vaccines and the mechanism by which mRNA vaccines induce anti-tumor immune responses, describe the current research and clinical applications of mRNA vaccines in tumor-targeted therapy, and forecast the future development trends of mRNA vaccine application in tumor-targeted therapy.

## Background

**Fig. 1:**
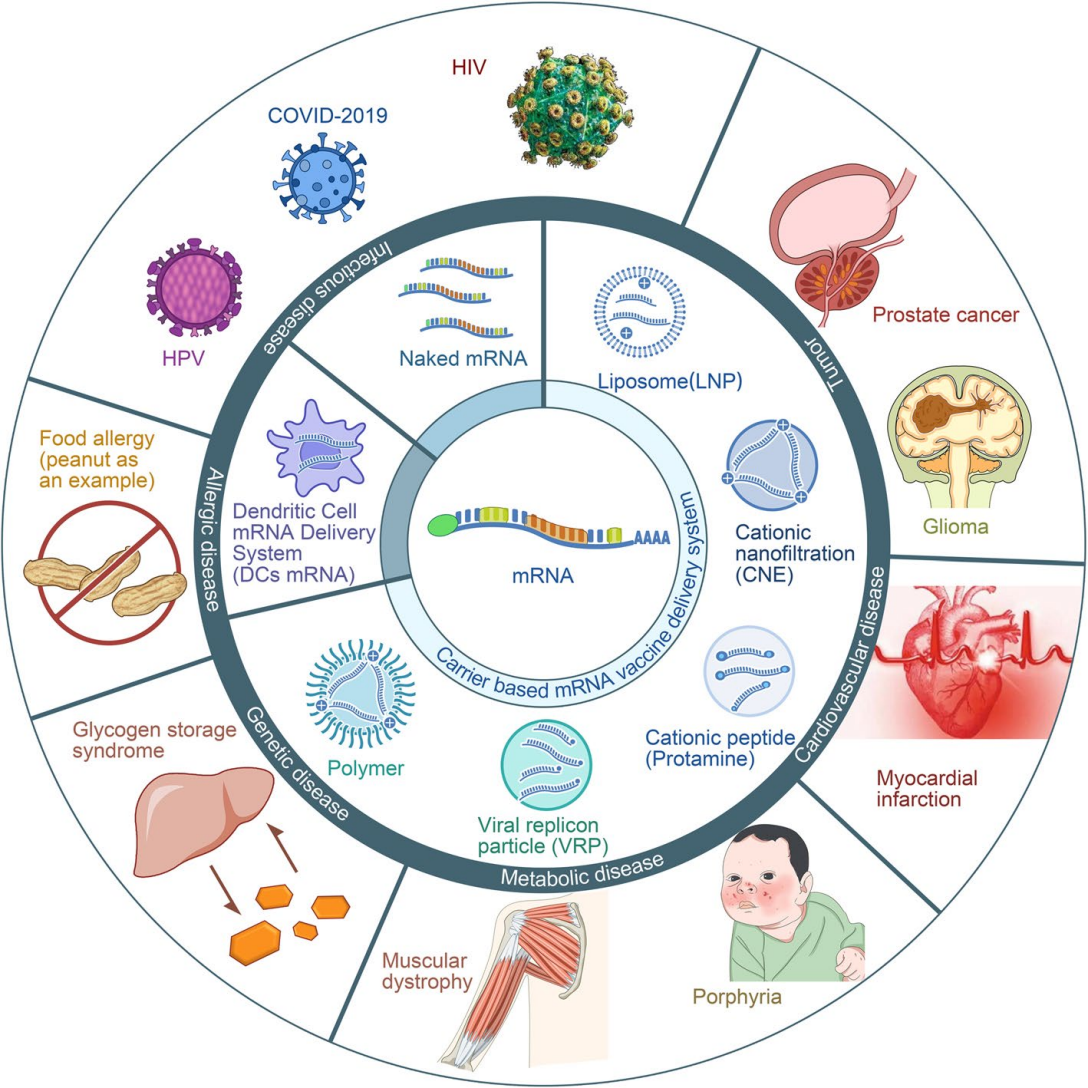
Application field of mRNA vaccines. Legend: The mRNA vaccine delivery systems primarily encompass three categories: 1) Carrier-based delivery systems, including lipid nanoparticles (LNPs), cationic nanoemulsions (CNEs), cationic peptides (e.g., protamine), viral replicating particles (VRPs), and polymers. 2) Dendritic cell mRNA delivery systems (DCs mRNA). 3) Naked mRNA. Presently, mRNA vaccines are predominantly employed in the treatment of various diseases, such as: 1) Cardiovascular diseases, including myocardial infarction and heart failure [6]. 2) Metabolic diseases, such as muscular dystrophy [7] and porphyria [8]. 3) Genetic disorders, including glycogen storage disease [9]. 4) Allergic diseases, such as food allergies [27]. 5) Infectious diseases, including human papillomavirus (HPV) [28], Corona Virus Disease 2019 (COVID-19) [29], and human immunodeficiency virus (HIV) [30], among others. 6) Tumors, such as prostate cancer [10] and glioma [31], among others. Naked mRNA vaccines are primarily utilized in the treatment of tumors [32] and infectious diseases [33]. DC-loaded mRNA vaccines are mainly applied in the treatment of tumors [10]

Although there have been considerable advancements in cancer treatment, malignant tumors still remain a primary cause of human mortality [1]. Conventional modalities such as surgery, chemotherapy, and radiotherapy remain widely utilized. Additionally, immune checkpoint inhibitors (ICIs) have pioneered novel avenues in tumor-targeted therapy, showing efficacy across diverse malignancies [2]. The growing landscape of tumor-targeted therapy offers hope to cancer patients. This innovative modality enables precise tumor cell targeting, minimizes damage to normal tissues, is tolerable at high doses, and demonstrates significant therapeutic efficacy [3]. mRNA vaccines represent a novel technology at the intersection of molecular biology and immunology and is at the forefront of gene therapy (Table 1). In these vaccines, exogenous antigens encoded by mRNA are introduced into somatic cells, promoting the synthesis of antigenic proteins. This concurrent activation of the body’s principal immune mechanisms, namely, cellular and humoral immunity [4, 5], underscores the pivotal role of mRNA vaccines in tumor-targeted therapy. In recent decades, great strides in experimental techniques have catalysed the widespread utilization of mRNA vaccines across diverse domains, with an emphasis on tumor-targeted therapy. Currently, mRNA vaccines are used for the treatment of various diseases, yielding favourable outcomes [6–10]. (Fig. 1). This review comprehensively discusses the targeted delivery mechanisms of mRNA vaccines and their pivotal role in tumor-targeted therapy. It explores in detail the processes by which mRNA vaccines activate the immune system to recognize and attack tumor cells. Furthermore, this review explores the role of mRNA vaccines in modulating the tumor microenvironment, emphasizing their potential to enhance anti-tumor efficacy by optimizing the working conditions of immune cells. A thorough assessment of the research and application progress of mRNA vaccines in tumor-targeted therapy is presented, including numerous clinical trials that demonstrate their actual effectiveness and potential across multiple cancer types. Finally, this review anticipates future trends in this field, which are expected to further advance the development of mRNA vaccines in cancer treatment. Through this review, we aim to provide readers with a comprehensive and in-depth perspective that aids in understanding the central role of mRNA vaccines in cancer therapy and their promising future prospects.

**Table 1:** Breakthroughs of mRNA vaccines in research

Year	Breakthrough in mRNA vaccine research	References
1990	Concept proposal of mRNA vaccines	[11]
1995	mRNA tested as cancer vaccine (in mice)	[12]
2000	Exploration of mRNA as a novel vaccine approach	[13]
2002	The first clinical trial with ex vivo DCs transfected with mRNA against cancer	[14]
2006–2008	mRNA modifications to enhance stability and efficacy, leading to improved performance and longevity in vaccine applications and therapeutic interventions	[15–18]
2010	Significant Progress of mRNA technology in infectious disease vaccines	[19]
2012	Intranodal delivery of mRNA transfects DCs and elicits anti-tumor immunity	[20]
2013	Debate on type I IFN in efficacy and safety of mRNA vaccines	[21]
2020–2022	FDA approval of two mRNA vaccines of COVID-19	[22, 23]
2022 to present	Research and clinical trials on personalized mRNA cancer vaccines	[24–26]

## Overview of mRNA vaccines: principle, classification, synthesis and biomarkers

mRNA vaccines are based on the "central dogma" of molecular biology and involve the optimization, chemical modification, and purification of mRNAs with specific antigens [34]. There are two main types of mRNA vaccines: self-amplifying (SAM) and nonreplicating vaccines [35]. SAM vaccines alter a virus's genome to include mRNAs encoding antigens, allowing self-replication without viral protein synthesis and increasing safety and efficiency. Nonreplicating vaccines contain only full-length mRNAs encoding the antigen, with a 5' cap structure and 3' poly(A) tail. Despite their simple structure and direct antigen focus, they have the drawbacks of a short half-life and low in vivo antigen expression [36], necessitating higher doses for effectiveness. The generation of designed DNA templates into an RNA strand is guided by the principle of base complementarity [37]. This process is accomplished through in vitro transcription (IVT), which involves sequence construction, IVT, capping, and tailing, is the primary method for synthesizing mRNA vaccines [38]. DNA templates for IVT must have an open reading frame (ORF), a 5' UTR and 3' UTR, and for self-amplification, a long ORF. The ORF contains start and stop codons [39], allowing splicing for mature mRNA production. The 5' UTR and 3' UTR regulate mRNA stability and translation [40]. The primary challenge facing IVT mRNAs is their immunogenicity. To address this issue, modifications using nucleotides can increase RNA stability and minimize immunogenicity. Among various nucleotide modification methods, chemical alterations, poly(A) tail addition, and sequence optimization are commonly employed [15]. Furthermore, mRNA purification is crucial for eliminating immunogenic properties [41]. Purification techniques mainly include different chromatographic methods (e.g., high-performance liquid chromatography (HPLC) [42], ion exchange [43], size exclusion [44], affinity [45], and Fast protein liquid chromatography (FPLC) [46]), adsorption [47], and membrane technology [41]. The cap structure shields mRNA from exonuclease degradation, ensuring mRNA stability and enhancing translation efficiency [48]. Methylation can be utilized to cap mRNAs in three primary forms: Cap0, Cap1, and Cap2. Traditional enzymatic capping is achieved by enzymatic capping [49], which involves RNA 5'-triphosphatase (RTPase) hydrolysing the 5′ end of RNA, which transfers guanosine monophosphate (GMP) via guanylyltransferase (GTase) to form a cap structure (m7GpppNp), which can be further modified to cap1 (m7GpppN1mp) or cap2 through 2'-O-methyltransferase. Cotranscriptional capping with a Cap analogue (m7GpppG) is also a common method [50] used during mRNA transcription [51]. However, studies have indicated that capping analogues may disrupt mRNA binding, affecting translation efficiency by hindering ribosome recognition and proper 5' end determination [52, 53]. Adding a poly(A) tail is essential for ensuring the stability of mRNA after transcription [54]. There are two main methods used for adding poly(A) tails to mRNAs. The first involves traditional enzymatic polyadenylation, in which the poly(A) tail is added to the 3' end of the mRNA without changing the length of the tail [55]. The other method involves obtaining a poly(A) tail of controllable length by formatting a fixed-length poly(A) sequence on the basis of a DNA template and transcribing it [56]. The ideal length of the poly(A) tail falls within the range of 120 to 150 nucleotides [57–59]. Research on biomarkers for mRNA vaccines remains limited, encompassing two primary areas: 1) Immunophenotyping. Studies have indicated that immunophenotyping can reflect the expression levels of immune checkpoint (ICP) and immunogenic cell death (ICD) regulators, suggesting its potential as a therapeutic biomarker for mRNA vaccines [60]. 2) Tumor antigens. Lin et al. identified six genes that may serve as vaccine targets and stimulate antigen-presenting cell (APC) activation in glioblastoma (GBM), suggesting that these genes are potential biomarkers for mRNA vaccines [61]. Another study demonstrated that patients with malignant mesothelioma (MESO) characterized by high expression of the oncogene fibronectin 1 (FN1) may develop resistance to mRNA vaccination. Consequently, the authors suggest that FN1 could serve as a potential biomarker for mRNA vaccines [62]. However, these studies are primarily bioinformatics analyses, and further exploration through in vitro and in vivo studies is necessary to elucidate the biomarkers associated with mRNA vaccines.

## Targeted delivery mechanism of mRNA vaccines

A reliable and secure targeted delivery mechanism is highly important for the progress of mRNA vaccine technology [63]. Currently, several mRNA delivery systems are known, including, Carrier based delivery system, naked mRNA and the dendritic cell-mRNA delivery system (DCs-mRNA) [64](Table 2).

**Table 2:** mRNA vaccine delivery systems

Delivery types	Delivery subtypes	Advanntages	Challenges
Carrier based delivery system	Liposomes and their derivatives, mainly lipid nanoparticles(LNPs) [65]	Lipid nanoparticles (LNPs) demonstrate a remarkable mRNA encapsulation efficiency, which is pivotal for the protection of mRNA from nuclease degradation and subsequent stable delivery to the target cells Moreover, LNPs possess a distinct advantage in terms of tissue penetration, which facilitates deeper penetration into tissues and organs, thereby enabling more widespread and efficient cellular uptake. The nanoscale dimensions of LNPs contribute to their enhanced intracellular delivery, as they can easily traverse cellular barriers and accumulate within the target cells In addition to their delivery efficiency, LNPs exhibit low cytotoxicity and immunogenicity, which are critical attributes for their application in therapeutic settings Another notable feature of LNPs is their potent adjuvant properties, which are essential for enhancing the immune response when delivering vaccines or immunotherapies [66–68].	Lipid nanoparticles (LNPs) are susceptible to degradation, showcasing suboptimal stability under storage conditions, with a propensity for aggregation and fusion phenomena, which can compromise their structural integrity and therapeutic efficacy [69].
	Polymers [70]	Certain polymers have demonstrated the ability to significantly enhance the process of endosomal escape, thereby improving the delivery efficiency of therapeutic agents. Additionally, these polymers provide protection for messenger RNAs (mRNAs) against enzymatic degradation, ensuring their stability, and facilitate a safe and effective release of mRNAs into the cytoplasm for subsequent translation [71].	The low purity and high molecular weight of polymer-based delivery vectors, coupled with their high charge density, can result in significant cytotoxicity [72]
	Virus-like replicon particles [73]	Viral replicon particles (VRPs) have the unique capacity to encapsulate self-amplifying RNA (saRNA)-encoded antigens, effectively facilitating their transport to the cytosol. Through in vitro synthesis, viral structural proteins can be produced and utilized for encapsulating saRNAs that encode specific antigens. Extensive researches [74] has illuminated the therapeutic potential of mRNA vaccines administered via VRPs across a diverse array of viral, bacterial diseases, and cancer. This method enhances RNA replication, elicits potent innate immune responses, and promotes the maturation of dendritic cells, contributing to the vaccines' efficacy and immunogenicity.	Viral replicon particles (VRPs) possess a notable disadvantage, as they have been observed to elicit neutralizing antibody responses specifically targeted against the viral surface proteins, as evidenced by studies [75, 76].
	Cationic nanoemulsion (CNE) [77]	CNE can enhance the efficacy of mRNA vaccines by binding to saRNA in a pH-dependent manner, comprising nanoemulsions and cationic lipids. Nanoemulsions can be generated via techniques such as ultrasound, microfluidics, and vigorous stirring [78]. Among the CNE components, the cationic lipid 1.2-diol sn glycerol-3-phosphate choline (DOTAP) stands out for its positive charge, being emulsified with MF59, the identical adjuvant component of the lotion [79]. Additionally, CNE has shown promising therapeutic effects in its ability to deliver saRNA, indicating that lower doses of adjuvant subunits in CNE complexes can elicit substantial immune responses [80]. Numerous studies have been conducted to investigate the stability, toxicity, and biodistribution of CNE, with findings confirming its stability [81].	However, the conclusions regarding the toxicity of CNE vary across different models. One study demonstrated that the toxicity of nanoemulsions on human foetal lung cells (MRC-5) is dose-dependent [82]. In contrast, another investigation revealed that the rabies animal model exhibited suitable tolerance to CNE-delivered self-amplifying mRNA (SAM) vaccines [83]
	Cationic cell-penetrating peptides (CPP) [84, 85]	Cationic peptides, including protamine, a well-established cationic peptide utilized for mRNA transport [86], facilitate the formation of nanosized complexes with mRNAs. These complexes effectively shield the mRNA from enzymatic degradation, maintain immunogenicity across varying temperatures, and preserve the potency of antigen-encoded mRNA vaccines [87]. Protamine's ability to spontaneously condense mRNA via electrostatic interactions serves to protect the enclosed mRNA from degradation by extracellular RNases [88, 89]. Furthermore, the protamine-mRNA complexes demonstrate adjuvant properties, stimulating TLR7/8 to trigger robust innate immune responses [90].	The specific combination ratio and binding strength between protamine and mRNA are crucial factors that can significantly influence the translation process. These parameters may impose limitations on the efficiency of vaccine protein expression, ultimately affecting the overall effectiveness of the vaccine in eliciting an immune response and providing protection [91].
Naked mRNA	-	First, the mRNA cannot be integrated into the genome, reducing the risk of genetic mutations. Second, ribosomes can bind directly to the mRNA in the cytoplasm, causing the mRNA to be translated immediately and rapidly initiating an immune response after vaccination. Third, the final position of the mRNA determines the site of protein expression, allowing for precise control of protein expression [92, 93].	The lack of a carrier during the delivery process can lead to unstable protein translation and expression. However, this can be mitigated by altering the administration method and proper chemical modifications. However, research in this area is relatively limited at present [94].
Dendritic Cell-mRNA Delivery System (DCs-mRNA)	-	Dendritic cells (DCs) serve as the orchestrators of the immune response, exhibiting unparalleled efficiency in their ability to capture and present antigens. This is achieved through a meticulously regulated process involving internalization and proteolytic degradation. Following this intricate mechanism, DCs proceed to present antigens to CD8 + T or CD4 + T cells via major histocompatibility complexes (MHCs), specifically MHC class I (MHCI) or MHC class II (MHCII). By doing so, they initiate an adaptive immune response [95]. The pivotal role of DCs in this context underscores their significance as prime targets for vaccination strategies.	Challenges primarily include the two aspects: Firstly, serum protein aggregation and mRNA degradation upon systemic administration [96], compromising vaccine integrity. Additionally, the second challenge involves the efficient systematic dissemination of mRNA vaccines, ensuring uniform distribution [97] for optimal immune response.

### Carrier based delivery system

Two major types of carrier molecules have been utilized in nucleotide delivery systems: viral carriers and non-viral carriers [98]. However, owing to associated limitations such as potential immunogenicity, tumorigenicity, and low drug loading, the use of viral carriers has been limited. Conversely, nonviral carriers, including liposomes and their derivatives [65], polymers [70], virus-like replicon particles [73], cationic nanoemulsion (CNE) [77], and cationic cell-penetrating peptides (CPP) [84, 85], have garnered significant attention. Among these carriers, liposomes and their derivatives, particularly lipid nanoparticles (LNPs) [99, 100], stand out as widely employed delivery systems. LNPs typically have four key components: ionizable amino lipids, cholesterol, polyethylene glycol lipids, and auxiliary lipids such as double stearyl phosphatidylcholine (DSPC) [101–103]. LNPs, which are approximately 100 nm in diameter, are strikingly similar in both size and composition to various viral entities, mirroring the dimensions of infectious agents such as the SARS-CoV-2 virus (approximately 100 nm), influenza A virus (ranging from 80 to 120 nm), and mature HIV particles (approximately 100 nm in diameter) [104]. Post-administration, LNPs are dynamically transported to cells expressing lipid or scavenger receptors akin to natural apolipoprotein conveyance. LNPs offer notable advantages, including high delivery efficacy [105] and commendable biocompatibility. Polymer materials primarily feature cationic liposome polymers (LPPs) with a positive charge that are proficient in mRNA encapsulation to enable protein expression while mitigating degradation risks. However, these methods have limitations such as polydispersity and macromolecule elimination [106]. Lipid nanoparticles (LNPs) exhibit high mRNA encapsulation efficiency and effective cellular transfection, coupled with robust tissue penetration, low cytotoxicity and immunogenicity, and potent adjuvant properties [66–68]. However, LNPs are prone to degradation and exhibit relatively poor stability during storage, tending to aggregate and fuse [69]. Nevertheless, numerous preclinical and clinical trials have confirmed that LNPs hold promising potential as mRNA vaccine carriers, capable of effectively activating immune responses. Continuous technological advancements have led to LNPs with more complex structures and enhanced physical stability [107], yielding substantial achievements in the innovation of vaccine delivery systems [71]. LPPs encompass diverse materials like polyethyleneimine (PEI) [108], polyamide amine (PAMAM) dendritic polymer [109], dendritic macromolecular polypropylene imine [pol (propylene imine), PPI], polyurethane [poly (aminoester), PAE], and polysaccharides [110]. Polyethylenimine (PEI) has been shown to enhance endosomal escape, protect mRNAs from degradation, and facilitate safe release into the cytoplasm [111]. However, the low purity and high molecular weight of polymer-based delivery vectors, coupled with their high charge density, can result in significant cytotoxicity [72]. Cationic peptides, characterized by cations or amphiphilic amino groups (e.g., arginine) in the main and side chains, facilitate mRNA delivery. Notably, protamine, a renowned cationic peptide for mRNA transport [86], forms nanosized complexes with mRNAs to safeguard against RNA enzyme degradation and stabilize immunogenicity across temperatures while preserving the efficacy of antigen-encoded mRNA vaccines [87]. Protamine can spontaneously condense mRNA through electrostatic interactions, thereby protecting the encapsulated mRNA from degradation by extracellular RNases [88, 89]. Additionally, protamine-mRNA complexes can function as adjuvants, activating TLR7/8 to elicit innate immune responses [90]. The combination ratio and binding strength between protamine and mRNA can have implications for the translation process, potentially limiting vaccine protein expression efficiency and overall vaccine effectiveness [91]. Viral replicon particles (VRPs) have the capacity to encapsulate self-amplifying RNA (saRNA)-encoded antigens and facilitate their transportation to the cytosol. In vitro synthesis of viral structural proteins allows for their encapsulation as saRNAs encoding specific antigens. Numerous studies have highlighted the therapeutic potential of mRNA vaccines delivered via VRPs against a spectrum of viral diseases, bacterial diseases, and cancer [74]. It enhances RNA replication, triggers innate immune responses, and promotes the maturation of dendritic cells. However, it also has the drawback of inducing neutralizing antibody responses against the viral surface [75, 76]. CNE can enhance the efficacy of mRNA vaccines by binding to saRNA in a pH-dependent manner, comprising nanoemulsions and cationic lipids. Nanoemulsions can be generated via techniques such as ultrasound, microfluidics, and vigorous stirring [78]. Notably, among the CNE components, the cationic lipid 1.2-diol sn glycerol-3-phosphate choline (DOTAP) stands out for its positive charge, being emulsified with MF59, the identical adjuvant component of the lotion [79]. Additionally, a preclinical study conducted by Brito et al. on the ability of CNE saRNA delivery in rabbits, mice, and nonhuman primates revealed promising therapeutic effects of CNE and indicated that lower doses of adjuvant subunits in CNE complexes could elicit substantial immune responses [80]. Numerous studies have been conducted to investigate the stability, toxicity, and biodistribution of CNE, with findings confirming its stability [81]. However, the conclusions regarding its toxicity vary across different models. One study demonstrated that the toxicity of nanoemulsions on human foetal lung cells (MRC-5) is dose-dependent [82]. In contrast, another investigation revealed that the rabies animal model exhibited suitable tolerance to CNE-delivered self-amplifying mRNA (SAM) vaccines [83]. Biomimetic carriers represent an innovative drug delivery concept employing endogenous substances, biological structures, and processes. Exosomes, a type of lipid bilayer microvesicle characterized by small size and low immunogenicity, are a particularly auspicious biomimetic carrier. Exosomes can prolong the duration of drugs in circulation by evading mononuclear phagocytic system clearance, thereby increasing drug delivery efficiency [112]. In addition, promising new materials for mRNA vaccine delivery research, such as inorganic nanomaterials and hydrogels, are also being explored. Compared with traditional materials, these new materials have great advantages in terms of improving the efficiency and intensity of vaccine mRNA translation [113].

### Naked mRNA

Naked mRNA delivery refers to the direct administration of mRNA. This technology has been successfully used in vivo for immune responses, specifically targeting antigen-presenting cells through intradermal [92, 114] and intranodular injections [115, 116]. There are many advantages associated with this delivery method [92, 93]. First, the mRNA cannot be integrated into the genome, reducing the risk of genetic mutations. Second, ribosomes can bind directly to the mRNA in the cytoplasm, causing the mRNA to be translated immediately and rapidly initiating an immune response after vaccination. Third, the final position of the mRNA determines the site of protein expression, allowing for precise control of protein expression. Despite its advantages, naked mRNA delivery also has some major drawbacks [94]. The lack of a carrier during the delivery process can lead to unstable protein translation and expression. However, this can be mitigated by altering the administration method and proper chemical modifications. However, research in this area is relatively limited at present.

### Dendritic Cell-mRNA Delivery System (DCs-mRNA)

DCs are the orchestrators of the immune response, showing unparalleled efficiency in capturing and presenting antigens through a meticulously regulated process of internalization and proteolytic degradation. Subsequently, DCs present antigens to CD8 + T or CD4 + T cells through major histocompatibility complexes (MHCs), specifically MHCI or MHCII, thereby initiating an adaptive immune response [95]. This highlights DCs as prime targets for vaccination. Typically, specific mRNAs encoding antigens are delivered into DCs via electroporation, lipid transfection, nuclear transfection, or in vitro acoustic evaporation. Among these techniques, electroporation is preferred [24]due to its high transfection efficacy and independence from carrier molecules. Challenges primarily include the following two aspects: Firstly, serum protein aggregation and mRNA degradation upon systemic administration. Experts suggest addressing this issue by supplementing DCs with granulocyte–macrophage colony-stimulating factor (GMCSF) and IL-4 [96]. The second challenge lies in the systematic dissemination of mRNA vaccines [97].

## Mechanism of mRNA Vaccine-Induced anti- tumor immune response

The mechanisms by which mRNA vaccines induce an anti-tumor immune response involve two primary mechanisms. First, they directly induce tumor-specific T-cell responses, including both innate and adaptive immune responses. During this process, mRNA vaccines deliver tumor-associated antigens or tumor-specific antigens generated by intratumoral mutations to the immune system, activating antigen-presenting cells (APCs) and T cells and thereby initiating a specific antitumor immune response. Second, they achieve induction of an antitumor immune response by modulating the tumor microenvironment.

### Key factors in the induction of anti- tumor immune response by mRNA vaccines

The key factors in the mRNA vaccine-induced antitumor immune response include the following aspects. First, the design of the vaccine is crucial [117]. By precisely selecting tumor-associated antigens, a vaccine can ensure high specificity and effectiveness. Second, efficient antigen presentation is a key step in inducing an anti-tumor immune response [118]. mRNA vaccines express tumor-associated antigens, making them targets antigen-presenting cells (APCs) in vivo and thereby enhancing antigen presentation efficiency [119]. Third, a refined immune regulatory mechanism contributes to achieving immune balance [120]. mRNA vaccines can induce the generation of immunosuppressive cells and inflammatory factors to balance the immune response, preventing excessive immune damage [121].

### Molecular mechanisms underlying the activation of anti- tumor immune response by mRNA vaccines

The molecular mechanisms underlying mRNA vaccine-induced activation of anti-tumor immune responses involve multiple factors, including antigen presentation, immune cell activation, immune regulation, and antigen stimulation of B cells [122].

#### Inducing innate immunity

**Fig. 2:**
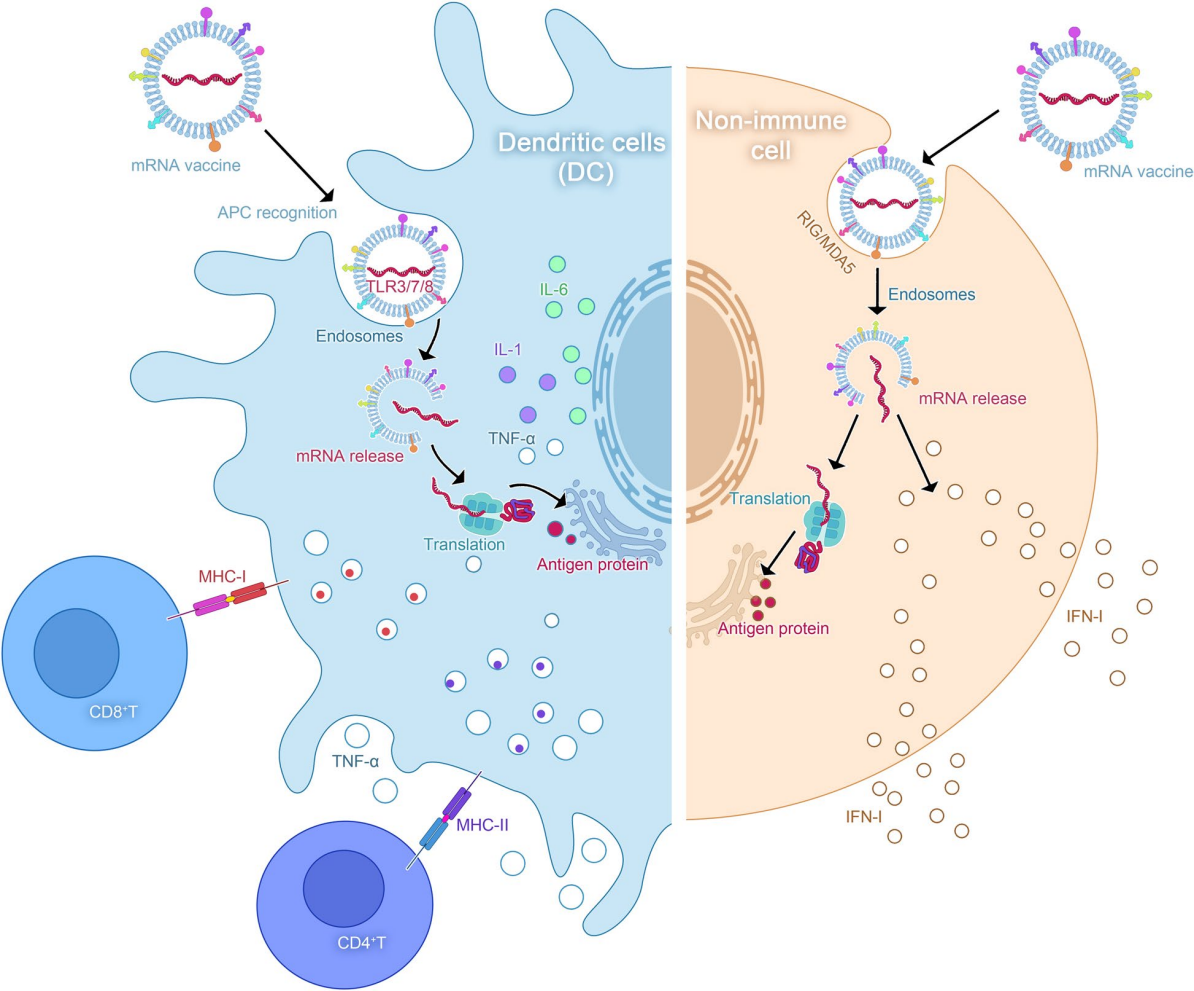
mRNA vaccine induces innate immune mechanism. Legend: Upon stimulation of DC cells, the T cells undergo identification, whereby the MHC complex and TCR receptor on their surface serve as the initial signals for cellular immune response. Antigen-presenting cells (APCs) recognize the mRNA, activating TLR and prompting the detection of PAMP, thereby initiating the second signal. The activated second signal translocates to the nucleus as a transcription factor, recruiting various Trans-acting factors to facilitate the expression of proinflammatory cytokines and chemokines. This dual signal pathway effectively activates the initial T cells. In non-immune cells, RIG-I and MDA5 are involved in sensing exogenous mRNA and inducing cytokines/chemokines to recruit innate immune cells

Congenital immune stimulation is driven primarily by the response mechanism of immune cells in defence against pathogens. The Golgi apparatus and endoplasmic reticulum cooperate to translate protein fragments via the MHC [123]. Following DC activation, the MHC can identify corresponding T cells and initiate cytotoxic lymphocyte immunity [124]. MHC complexes and TCRs found on the surface of T cells are the first signals that trigger cellular immune responses. Several components of mRNA vaccines can interact with pattern recognition receptors (PRRs) in endosomes, including TLR3/7/8, which can detect pathogen-associated molecular patterns (PAMPs) in mRNAs. TLR7/8 recognizes single-stranded RNA (ssRNA), whereas TLR3 detects double-stranded RNA (dsRNA). APCs can identify mRNAs and activate TLRs [125]. The activated TLR detects PAMPs and triggers the second signal. The activated second messenger translocates to the nucleus and functions as a potential transcription factor. It can recruit various transactivating factors to promote the expression of proinflammatory cytokines and chemokines such as interleukin-6(IL-6), interleukin-2(IL-2), and tumor necrosis factor-α (TNF-α), thereby activating naive T cells via dual signalling pathways. However, there may be insufficient T lymphocytes to initiate cellular immune responses. Therefore, when these stored cells are exposed to the same antigen again, they are quickly activated. The proper absorption of APCs is a prerequisite for the activation of an immune response, with DCs being primarily responsible. A previous study [126] showed that mRNA vaccines have the ability to stimulate DC cell maturation. In non-immune cells, RIG/MDA5 recognize exogenous mRNA, activating cytokine and chemokine production [127]. Subsequently, they are able to recruit innate immune cells (Fig. 2).

#### Inducing adaptive immunity

**Fig. 3:**
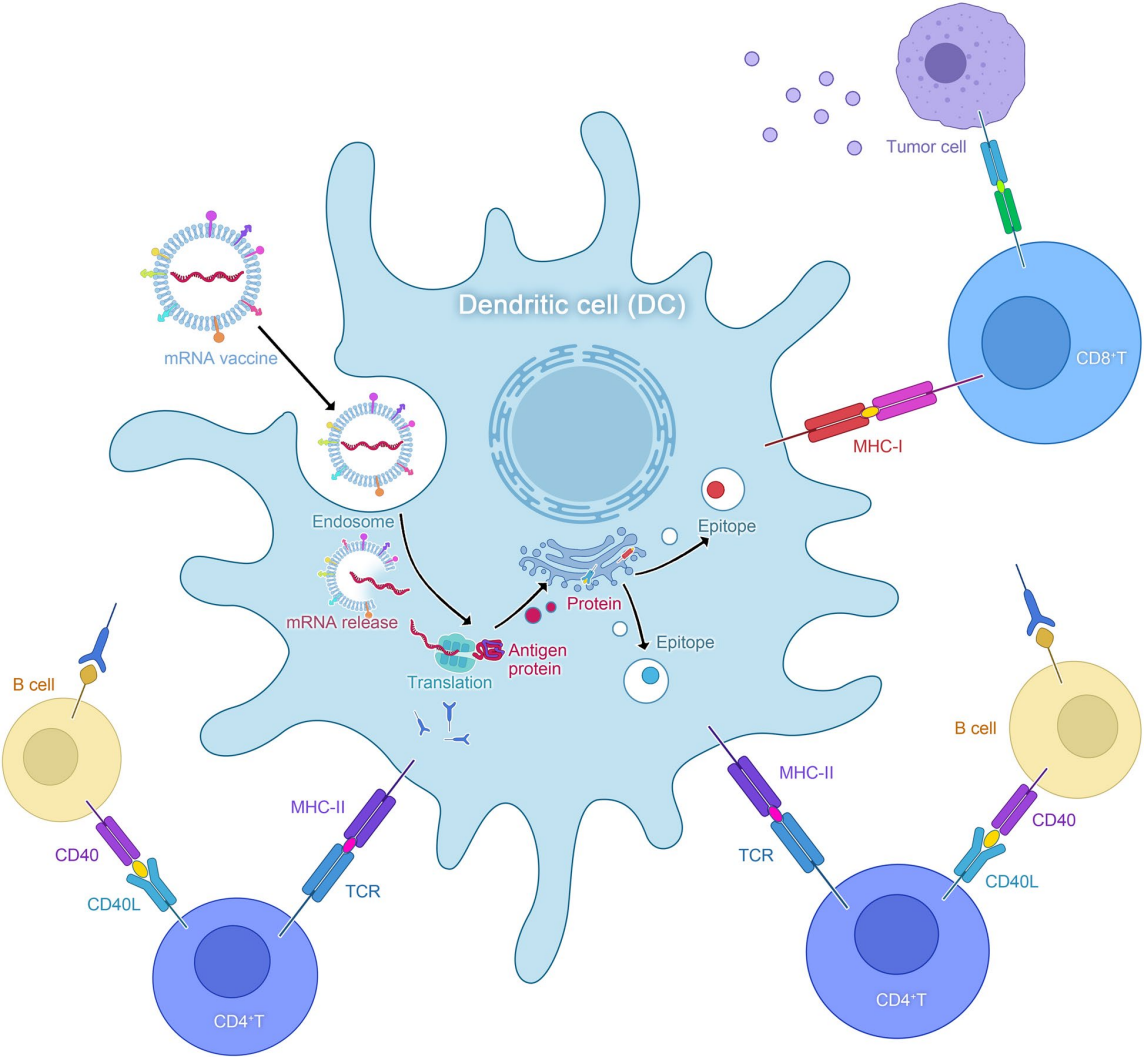
mRNA vaccine induces adaptive immune mechanism. Legend. After translation, the proteins encoded by mRNA are taken up by antigen-presenting cells (APCs) via mechanisms such as micropinocytosis, endocytosis, or phagocytosis. These antigens are subsequently processed into peptides and loaded onto the MHC class I pathway. The translation conducted by ribosomes produces immunogenic proteins, which are degraded into fragments within the proteasome and presented to CD8 + T cells via MHC-I. An alternative pathway allows for the direct transport of antigens from the cytoplasm to lysosomes, or the incorporation of a lysosome-targeting sequence within the mRNA structure for lysosomal degradation. The resulting MHC-II peptide complexes are then recognized by the T cell receptor (TCR) on CD4 + T cells

After translation, APCs, such as DCs, take up the protein encoded by mRNA by a variety of mechanisms including micropinocytosis, endocytosis, or phagocytosis [128]. Antigens can be transiently expressed and accumulate in the cytoplasm, allowing for rapid processing into peptides that can be recognized by MHC I. Ribosomal translation generates various antigenic proteins, which are then degraded into fragments in proteasomes and presented as CD8 + T-cell epitopes by MHC-I. Alternatively, antigens can also be transported directly from the cytoplasm to lysosomes, or lysosomal-targeting sequence antigen proteins can be incorporated into mRNA structural design, followed by lysosomal disintegration and presentation as CD4 + T-cell epitopes by MHC-II. In summary, APCs can present exogenous antigens to CD4 + T cells through MHC-II while also cross-presenting exogenous antigens to CD8 + T cells via MHC-I, resulting in the activation of cytotoxic T cells. This stimulation method is termed cross-stimulation. CD4 + T cells can provide support to other immune cells, including B cells and CD8 + T cells, through their helper functions. Ultimately, the cloning amplification of alloantigen-specific T and B cells can result in the elimination of target cells. Furthermore, all nucleated cells possess the ability to process mRNA and present various translated proteins as well as peptides in the MHC-I pathway. Among them, only APCs can present on both MHC-I and MHC-II, triggering immunological responses from CD4 + T or B cells. However, prior to activating adaptive immunity, understanding how cells recognize non-self mRNAs and activate signaling cascades through the interplay of mRNAs, PRRs, and PAMPs is critical. PRRs that can perceive these PAMPs are mainly categorized into two distinct types: extracellular and intracellular [129]. PRRs that recognize RNA contribute to the production of IFN-I. Furthermore, IFN-γ can stimulate the activation of Protein Kinase R (PKR) and eIF2α phosphorylation, leading to cellular and humoral immune responses. As a result, IFN-γ is expected to provide immunological protection to the body. However, mRNA vaccines can overstimulate the immune response, causing excited cells to generate a significant amount of IFN-I, which inhibits mRNA translation and promotes mRNA degradation. Consequently, this downregulates the expression of the target protein, causing a negative reaction on the immune response. Therefore, an effective mRNA vaccine should completely activate innate immunity before initiating adaptive immunity (Fig. 3).

### Modulation of the tumor microenvironment by mRNA vaccines

Based on definition provided in a previous report [130], the tumor microenvironment can be described as a local inner environment composed of tumor-infiltrating immune cells, interstitial cells, and active mediators released by these cells along with tumor cells. This microenvironment is critical for tumor growth and progression because it provides important nutrients and energy while also assisting tumor cells in evading immune system responses. Furthermore, the tumor microenvironment has been shown to increase the propensity of tumors to metastasize to other parts of the body. mRNA vaccines, as novel strategies for cancer immunotherapy, also greatly affect the tumor microenvironment [131].

#### The role of tumor microenvironment during tumor progression

The tumor microenvironment is a complex and dynamic ecosystem within tumor tissue that consists of a diverse array of components, such as tumor cells, immune cells, fibroblasts, extracellular matrix proteins, and an intricate network of cytokines and chemokines. These elements interact in a highly regulated manner, playing crucial roles in tumor growth, invasion, immune evasion, and response to therapy. Understanding the interactions within the tumor microenvironment is essential for developing effective strategies for cancer treatment and improving patient outcomes [132]. For example, cancer-associated fibroblasts (CAFs) and other immune cells have been reported to contribute to this process [133].The tumor microenvironment provides a favorable habitat in which the tumor cells can rapidly proliferate, evade immune surveillance, and metastasize [134]. Tumor cells adapt and evade the immune system by modulating immune suppression signals in response to antitumor immunological pressure [135]. Ultimately, tumor cells create an immunosuppressive microenvironment, which can enhance anti-tumor immunity and promote tumor survival. Thus, an ideal mRNA vaccine may alter the composition of local immune cells while restoring tumor immune surveillance.

#### mRNA vaccines can alter the distribution of cytokines in the tumor microenvironment

First, mRNA vaccines can alter the levels of cytokines in the tumor microenvironment by expressing tumor-associated antigens, thereby influencing the activation of immune cells and inflammatory responses [136]. The expression of tumor-associated antigens activates immune cells, particularly CD4 + T cells and CD8 + T cells, prompting them to release more cytokines [137]. An increase in these cytokines can disrupt the balance between immunosuppressive cells and inflammatory factors in the TME, making it easier for immune cells to penetrate into tumor tissues and eliminate tumor cells [138]. mRNA vaccines can promote DC maturation through TLR signaling. mRNA vaccines activate the transcription factor NF-κB via the MyD88 and TRIF pathway, thus promoting the generation of cytokines such as interleukins(ILs), tumor necrosis factors(TNFs), and interferon(IFNs), as well as the maturation of cytotoxic T lymphocytes (CTLs), resulting in the elimination of solid tumors during tumor targeted therapy [139]. Furthermore, mRNA vaccines can promote cytokine release by helper T cells, thereby increasing the level of antibodies of the humoral dependent immunity [140]. Furthermore, mRNA vaccines can enhance the recruitment and activation of antigen-presenting cells (APCs) in the TME [141]. APCs, like dendritic cells, macrophages, and B cells, initiate immune responses by presenting tumor antigens to T cells through mRNA vaccines, activating specific anti-tumor immunity [142]. This process helps to establish a bridge between innate and adaptive immune responses in the TME, thereby enhancing anti-tumor immune activity. In addition, mRNA vaccines can also regulate the expression of immune checkpoint molecules in the TME [143]. Immune checkpoints are a type of immune inhibitory molecules that play a key role in regulating immune responses and maintaining self-tolerance [144]. mRNA vaccines can influence the expression of immune checkpoint molecules on immune cells and tumor cells, potentially overcoming the immune evasion mechanisms employed by tumors [28]. By intervening in immune checkpoints, mRNA vaccines can enhance anti-tumor immune responses and improve the efficacy of tumor immune therapy [119].

#### mRNA vaccines can modulate tumor immune microenvironment (TIME)

**Fig. 4:**
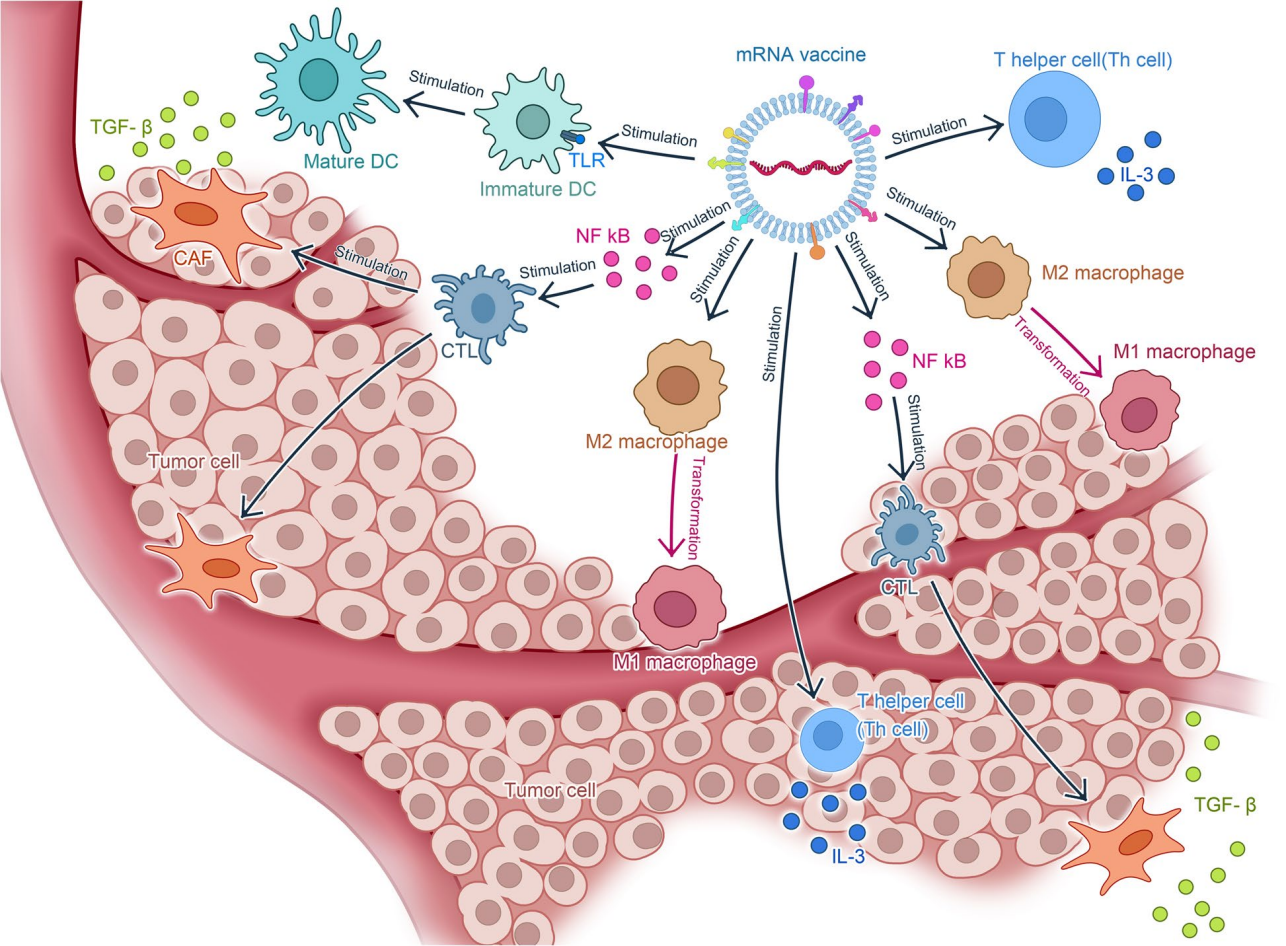
mRNA vaccines reshape tumor immune microenvironment (TIME). Legend.mRNAvaccine possesses the potential to reshape the tumor immune microenvironment via two primary mechanisms. Firstly, it regulates the equilibrium between M1 and M2 macrophages, thus transforming M2 macrophages into M1 macrophages. Secondly, it induces the secretion of cytokines by various T cells (For example T helper cell). Additionally, the vaccine promotes the maturation of dendritic cells (DC) through Toll-like receptor (TLR) receptors, activates the transcription factor NF kB to stimulate the maturation of cytotoxic T lymphocytes (CTL), and prompts T helper cells to secrete cytokines

mRNA vaccines have the potential to reshape the tumor immune microenvironment (TIME) via two primary mechanisms: 1) regulating the balance between M1 and M2 macrophages and 2) stimulating cytokine release by different types of T cells (Fig. 4). The interaction of malignant cells and immunological components in the tumor microenvironment (TME) has a great effect on tumor growth and maturation. Tumor cells frequently exploit immunosuppressive mechanisms, such as the production of immunosuppressive proteins, to evade immune surveillance. However, studies have shown that mRNA vaccines can restore tumor immunosurveillance by increasing MHC-I expression [145]. mRNA vaccines can also inhibit tumor growth by regulating the ratio of M1 to M2 macrophages to control tumor progression. Macrophages carry out their functions through two subtypes: M1 and M2. M1 macrophages promote inflammation, whereas M2 macrophages suppress it. In the tumor microenvironment, macrophages mostly exhibit the M2 phenotype. However, studies have shown that mRNA vaccines can increase the ratio of M1 macrophages to M2 macrophages by promoting the transformation of M2 macrophages into M1 macrophages. This is particularly useful for suppressing tumor growth and mitigating tumor immune escape [146].

## Research and current applications of mRNA vaccines in tumor targeted therapy

In the realm of tumor-targeted therapy, mRNA vaccines are utilized in two main ways: mRNA tumor vaccines directly target tumor cells, and increasing treatment effectiveness by combining mRNA vaccines with other tumor-targeted therapies, such as immune checkpoint inhibitors. The continual evolution of these strategies has revolutionized tumor-targeted therapy, presenting novel avenues to enhance treatment outcomes among cancer patients and illustrating the promising role of mRNA vaccines in combating cancer.

### mRNA tumor vaccines

Currently, mRNA tumor vaccines produced using IVT mainly target four distinct types of molecules: (1) encoding tumor-associated antigens (TAA), (2) encoding tumor-specific antigens (TSA), (3) encoding tumor-Associated Viruses.

#### mRNA vaccines encoding TAAs

TAAs are expressed in normal cells as well, but at relatively higher levels in tumor cells [147]. These antigens typically arise from abnormal differentiation or dysfunction of tumor cells, such as carcinoembryonic antigen (CEA), PRAME, NY-ESO-1, etc. [148–150]. Although TAAs are expressed to some extent in normal tissues, the significant upregulation of their expression in tumor cells allows the immune system to generate targeted immune responses [151]. The utilization of TAAs as targets for mRNA vaccines has already initiated clinical investigations in various solid tumors and haematologic malignancies. mRNA vaccines have the potential to be designed for TAAs that are selectively expressed in cancer cells. CA125 is a TAA in ovarian epithelial carcinoma, whereas AFP is a TAA in liver cancer. Several clinical trials have used mRNA vaccines targeting similar TAAs for therapy, including NCT00831467, NCT03164772, and NCT01995708 [24]. (Table 3).

**Table 3:** Clinical trials of mRNA vaccines encoding TAAs

Cancer type		NCT number	Drug administration	Phase	TAA type	Status	Delivery system	Result
Respiratory system tumors (mainly non-small cell lung cancer)	non-small cell lung cancer	NCT03164772	BI 1361849 (CV9202) + Durvalumab + / − Tremelimumab	I/II	EGFR	Recruiting	Protamine	Good tolerance, and most patients (84%) have found antigen specific immune responses [24]
		NCT00923312	mRNACV9201	I/II	EGFR	Recruiting	Protamine	Good tolerance and immune response detected after treatment; The median progression and overall survival time were 5 months and 10.8 months, respectively [86]
		NCT01915524	With local irradiation (with or without pemetrexed and with or without EGFR tyrosine-kinase inhibitor)	I	EGFR	Recruiting	Naked RNA	Detectableantigen-specificimmunity in 21 (84%) patients. One (4%) patient had partial response in combination with chemotherapy treatment, and 12 (46%) patients had stable disease [25]
Reproductive system tumors	ovarian cancer	NCT04163094	W_ova1 + carboplatin/paclitaxel	I	OVA-1	Recruiting	Naked RNA	Not published
	recurrent epithelialOC	NCT01334047	DC-006 vaccine (mRNA encoding hTERT, survivin)	I/II	hTERT	Recruiting	DC	Not published
	Penile Neoplasms Malignant	NCT03418480	BNT113 (HPV16 E6 and E7 oncoproteins)	I/II	E6/E7	Recruiting	Unkown	Not published
	ovarian cancer	NCT01456065	DCs loaded with TERT-mRNA and Survivin-peptide	I	TERT-mRNA and Survivin-peptide	Unknown	DC	Not published
Skin tumor (mainly melanoma)	melanoma	NCT02410 733	NY-ESO-1, tyrosinase, MAGE-A3, and TPTE	I	NY-ESO-1, MAGE-A3, tyrosinase, TPTE	Active, not publishedt recruiting	Lipid nanoparticles	Immune responses against a minimum of one tumourassociated antigen in 39 (75%) patients. mRNA vaccine with anti-PD-1 therapy: six (35%) patients had partial response and two (12%) had stable disease; mRNA vaccine monot publishedtherapy: three (12%) patients had partial response, and seven (28%) had stable disease [152]
		NCT04526899	BNT111 (NY-ESO-1, tyrosinase, MAGE-A3, and TPTE) + cemiplimab	II	NY-ESO-1, MAGE-A3, tyrosinase, TPTE	Recruiting	RNA-LPX	Good tolerance, strong CD4 + and CD8 + T cell immunity after treatment, combined with PD-1 inhibitors, achieving an objective response rate of 35% [153]
		NCT00940004	Dendritic cells electroporated with mRNA encoding gp100 and tyrosinase	I/II	gp100	Completed	DC	Not published
		*NCT01676779*	mRNA; b.TAAs: MAGE-A3, MAGE-C2, tyrosinase, gp100	II	NY-ESO-1, MAGE-A3, tyrosinase, TPTE	Completed	DC	Good tolerance (symptoms: transient local skin reactions, flu like symptoms, shivering after infusion), and may increase the one-year survival rate (71% in the treatment group, 35% in the control group) [154]
		*NCT01302496*	mRNA; b.TAAs: MAGE-A3, MAGE-C2, tyrosinase, gp100	II	NY-ESO-1, MAGE-A3. tyrosinase. TPTE	Completed	DC	12 out of 15 patients showed T cell stimulation response.Some patients have strong immune responses; Both single therapy and combination therapy can induce multifunctional CD8 + T cell responses, which may provide a benchmark for achieving the immune stimulation levels required for sustained clinical remission [26]
		*NCT01676779*	mRNA; TAAs: MAGE-A3, MAGE-C2, tyrosinase, gp100	II	mRNA; TAAs: MAGE-A3, MAGE-C2, tyrosinase, gp100	Completed	DC	Good tolerance (symptoms: transient local skin reactions, flu like symptoms, shivering after infusion), and may increase the one-year survival rate (71% in the treatment group, 35% in the control group)
		NCT01456104	Langerhans-type dendritic cells (a.k.a. Langerhans cells or LCs)	I	HLA-A	Completed	DC	Unknown
		NCT00978913	DCs transfected with hTERT, survivin and p53	I	hTERT, survivin and p53	Completed	DC	Unknown
		NCT00961844	Dendritic cells—transfected with hTERT-, survivin- and tumor cell derived mRNA + ex vivo T cell expansion and reinfusion + Temozolomid	I/II	hTERT-, survivin- and tumor cell derived mRNA	Terminated	DC	Unknown
		NCT00929019	Autologous dendritic cells electroporated with mRNA	I/II	HLA-A2	Terminated	DC	Not reported
		*NCT01302496*	mRNA; b.TAAs: MAGE-A3, MAGE-C2, tyrosinase, gp100s	II	MAGE-A3, MAGE-C2, tyrosinase, gp100s	Completed	DC	Among the 15 patients, 12 had T cell stimulation response; Some patients have strong immune responses; Both single therapy and combination therapy can induce multifunctional CD8 + T cell responses, which may provide a benchmark for achieving immune stimulation levels required for sustained clinical remission [24]
Urinarysystem tumor (mainly prostate cancer)	Prostate cancer	NCT04382898	BNT112 (PAP, PSA, and three undisclosed antigens) + cemiplimab	I/II	PAP, PSA, and three undisclosed antigens	recruiting	RNA-LPX	Not published
		NCT01817738	mRNA vaccine CV9104	I/II		Terminated	Protamine	Not published
		NCT01446731	DCs transfected with PSA, PAP, survivin and hTERT mRNA + docetaxel	II	PSA, PAP, survivin and hTERT	Completed	DC	Not published
		NCT02692976	DC loaded with protamine/mRNA encoding keyhole limpet hemocyanin (KLH) + DC loading with MHC I binding peptides, NY-ESO-1 and MUC1 PepTivator®	II	keyhole limpet hemocyanin (KLH)	Completed	DC	Not published
		NCT01197625	Dendritic cell vaccine	I/II	PSA	Active, not recruiting	DC	Not published
		NCT01153113	Human telomerase reverse transcriptase mRNA (hTERT mRNA) transfected dendritic cell	I/II	hTERT	Withdrawn	DC	Not published
		NCT00831467	CV9103:PSA, PSCA, PSMA, STEAP1	I/II	PSA, PSCA, PSMA, STEAP1	Completed	Protamine	Good tolerance and immunogenicity
		NCT00831467	CV9103 (mRNA encoding 4 PSAs, PSCA, PSMA, and STEAP1)	I/II	PSAs, PSCA, PSMA, and STEAP1	Completed	protamine-stabilized mRNA	well tolerated,prolonged patient survival
Blood System Cancer(leukemia mainly)	leukemia	NCT05000801	Dendritic cell vaccine	I	PSAs, PSCA, PSMA, and STEAP1	recruiting	DC	Not published
		NCT01686334	Dendritic cell vaccine	I/II	PSAs, PSCA, PSMA, and STEAP1	recruiting	DC	Not published
		NCT03083054	Autologous dendritic cells electroporated with WT1 mRNA	I/II	WT1	Not published	DC	Not published
		NCT00834002	Wilms Tumor Gene (WT1) mRNA-transfected autologous dendritic cell	I	WT1	Completed	DC	Not published
		NCT02649829	dendritic cell vaccination plus chemotherapy	I/II	WT1	Active, not recruiting	DC	Not published
		NCT01734304	DCs electroporated with mRNA encoding WT1, PRAME, and CMVpp65	I/II	WT1, PRAME, and CMVpp65	Completed	DC	Not published
		NCT00510133	GRNVAC1 (mRNA encoding human telomerase reverse transcriptase (hTERT) and a portion of the lysosome-associated membrane protein LAMP-1 (LAMP))	II	hTERT, LAMP-1 (LAMP)	Completed	DC	Not published
		NCT02528682	MiHA mRNA-loaded PD-L-silenced DC	I/II	WT1	Completed	DC	Not published
		NCT01686334	Autologous WT1 mRNA-electroporated DCs	II	WT1	Recruiting	DC	Not published
		NCT01995708	CT7, MAGE-A3, and WT1 mRNA-electroporated Langerhans cells (LCs)	I	CT7, MAGE-A3, and WT1	Active, recruiting	DC	safe and therapeutic with a slight adverse event [24]
		NCT03083054	Autologous dendritic cells electroporated with WT1 mRNA	I/II	WT1	Active, not recruiting	DC	Not published
		NCT00965224	mRNA encoding for Wilms’ tumor antigen WT1	II	WT1	Recruiting	DC	Not published
		NCT00514189	mRNA in AML cell lysate	I	WT1	Recruiting	DC	Not published
		NCT02405338	mRNA encoding WT1 and PRAME	I/II	WT1	Active, not recruiting	DC	Not published
Digestive System Cancer	Colorectal Cancer with Liver Metastases	NCT05533697	mRNA-4359 (mRNA encoding IDO and PD-L1)	I/II	IDO	Recruiting	Unknown	ongoing
		NCT00228189	CEA mRNA-loaded DCs	I/II	CEA	Completed	DC	Not published
Neurological tumors	Glioblastoma	NCT03688178	Cytomegalovirus pp65-LAMP + temozolomide, varlilumab, and Td	II		Recruiting	DC	Not published
		NCT00639639	Cytomegalovirus pp65-LAMP + autologous lymphocyte transfer and Td	I	pp65-LAMP	Completed	DC	Not published
		NCT04573140	Autologous total tumor mRNA and pp65 full length (fl) lysosomal associated membrane protein (LAMP) mRNA loaded DOTAP liposome vaccine administered intravenously (RNA loaded lipid particles, RNA-LPs)	I	LAMP	Recruiting	RNA-LPX	Not published
		NCT02649582	Dendritic cell vaccine + temozolomide chemotherapy	I/II	WT1	Recruiting	DC	No
		NCT01291420	WT1 mRNA-electroporated autologous dendritic cell	I/II	WT1	Unknown	DC	No
		NCT00961844	mRNA encoding hTERT, survivin, and tumor mRNA	I/II	hTERT	Recruiting	DCs loaded and ex vivo T cell expansion and reinfusion	Not published
		NCT02366728	Human CMV pp65-LAMP mRNA-pulsed autologous DCs	II	pp65-flLAMP	Active, not recruiting	DC	Not published
		*NCT03548571*	Dendritic cell immunization + Adjuvant temozolomide	II/III		Active,not recruiting	DC	Not reported
Other Cancers	unresectable/metastatic/recurrent head and neck cancer	NCT04534205	BNT113 (mRNA encoding E6/E7)	II	E6/E7	Active, not recruiting	LPX	Not published

#### mRNA vaccines encoding TSAs

During the carcinogenesis process, malignant cells develop somatic mutations, resulting in the expression of protein sequences that are not expressed in normal cells. Proteasomes can convert these proteins into peptides. Thereafter, the peptides produced can bind to MHC-I receptors and be recognized as new antigens by T-cell receptors. These novel antigens are distinct to each patient, representing tumor specificity and providing opportunities for tumor-targeted therapies [25, 155]. The specific process procedure involves removing a single tumor and identifying specific novel antigens via next-generation sequencing. The new antigens encoded by mRNAs are subsequently injected into the same patient, eliciting an immune response that can attack the tumor [156]. mRNA can encode several antigens, resulting in the presentation of many epitopes. mRNA can express multiple neoantigens, either as individual molecular forms or concatenated forms of multiple coding sequences. Some tumor types can produce a large number of novel antigens, and expressing multiple epitopes may stimulate T cell responses from a perspective of inducing a broad immune response. The most critical aspect of this therapy is verifying the precise immunogenic non-synonymous somatic mutation found in the patients' tumors and discovering new gene expression epitopes. Interestingly, in a clinical trial (NCT03394937), 20 postoperative (IIc, III, and IV) melanoma patients received an intranasal injection of non-formulated mRNA vaccine (ECI-006) [32]. The results indicated that patients tolerated the low-dose mRNA vaccination (600 μg) well and experienced a significant immunological response. No adverse reactions of level 3 or higher were noted [32]. In a second study cohort (NCT03394937), patients with metastatic melanoma in stable condition following conventional treatment for 3–12 months were given ECI-006 vaccination and standard anti-PD-1 treatment, but the results have yet to be announced [32]. Some clinical trials have reported persistent objective reactions in cancer patients following treatment without unmanageable toxic effects (NCT03323398, NCT03480152, etc.) [24, 26] (Table 4).

**Table 4:** Clinical trials of neoantigen mRNA vaccines

Cancer type		NCT number	Drug administration	Phase	Neoantigen	Status	Delivery system	Result
Respiratory system tumors (mainly non-small cell lung cancer)	non-small cell lung cancer	NCT03908671	Personalized mRNA vaccine encoding neoantigen	I	Individual tumour mutations	Not yet recruiting	LPP(liposubcutaneousplex)nanodelivery	Not published
		NCT03948763	mRNA-5671 (KRAS gene driver mutations) + pembrolizumab	I	KRAS gene driver mutations	Recruiting	Lipid nanoparticles	Not published
Skin tumor (mainly melanoma)	melanoma	NCT03897881	mRNA-4157 (personalised cancer vaccine encoding 20 different mutated neoepitopes) + With pembrolizumab	II	20 different mutated neoepitopes	Active,not recruiting	Lipid nanoparticles	Not published
		NCT03480152	(NCI)-4650, a mRNA-based, personalized cancer vaccine	I	Immunogenic T-cell epitopes derived from neoantigens	Terminated	Naked mRNA	Mutation-specific CD4 + and CD8 + T-cell responses against predicted neoepitopes in three (75%) of four patients.No objective clinical responses [24, 26]
		*NCT02035956*	An individualised tumour mutation signature with ten selected neoepitopes for each patient	I	Ten selected neoepitopes for each patient	Completed	Naked mRNA	Out of 125 selected new epitopes, 60% can stimulate T cell responses; Good tolerance to vaccination
		*NCT03480152*	NCI-4650	I/II	Immunogenic neoantigens	Active, not recruiting	LNP	safe with a slight adverse event
		*NCT03468244*	personalized mRNA tumor vaccine	NA	Individual tumour mutations	Recruiting	LPP	Not published
Urinary system tumor (mainly prostate cancer)	Prostate cancer	NCT03289962	BNT122 (personalised cancer vaccine encoding individual tumour mutations)	I	Individual tumour mutations	Active, not recruiting	RNA-LPX	Good tolerance when used in combination with atezumab; Inducing the release of pro-inflammatory cytokines and peripheral T cell responses in most patients [25]
Blood System Cancer(leukemia mainly)	leukemia	NCT03468244	Personalized mRNA vaccine encoding neoantigen	I	Individual tumour mutations	Recruiting	LPP (lipo subcutaneous plex) nanodelivery	Not published
		NCT04486378	BNT122 (personalised cancer vaccine encoding individual tumour mutations)	II	Individual tumour mutations	Recruiting	RNA-LPX	Not published
Digestive System Cancer	Colorectal Cancer with Liver Metastases	NCT04161755	BNT122 (personalised cancer vaccine encoding individual tumour mutations) With oxaliplatin, irinotecan, fluorouracil, leucovorin, and atezolizumab	I	Individual tumour mutations	Active, not recruiting	RNA-LPX	Not published
	Esophagus Cancer	NCT03480152	National Cancer Institute (NCI)-4650, a messenger ribonucleic acid (mRNA)-based, Personalized Cancer Vaccine	I/II	Individual tumour mutations	Terminated	Lipid nanoparticles	It has safety and immunogenicity, with a maximum tested dose of 0.39 mg and no serious side effects observed
	Colorectal Cancer	NCT04534205	BNT122 (personalised cancer vaccine encoding individual tumour mutations)	II	Individual tumour mutations	Recruiting	RNA-LPX	Not published
	Pancreatic cancer	NCT02316457	BNT-114 plus BNT-122 (personalised set of pre-manufactured non-mutated shared tumour-associated antigens plus a personalised cancer vaccine encoding individual tumour mutations)	I	Individual tumour mutations	Active, not recruiting	DC	Not published
	gastric cancer, esophageal cancer, and liver cancer	NCT05192460	neoantigen tumor vaccine	I	individual tumour mutations	Active, not recruiting	Unknown	Not published
	colonic neoplasms and colorectal neoplasms	NCT05456165	GRT-C901/GRTR902	II	Deoxyribonucleic acid (DNA) mutations present peptides	Recruiting	Chimpanzee adenovirus	Ongoing
Other Cancer	TNBC	NCT02316457	IVAC_W_bre1_uID and IVAC_M_uID	I	Tumor-specific mutations	Active, not recruiting	LPX	Ongoing

#### mRNA vaccines targeting tumor-associated viruses

Tumor viruses are a class of viruses capable of inducing and promoting carcinogenesis in host cells [157]. Currently, the main viruses associated with human tumors include hepatitis B virus (HBV), hepatitis C virus (HCV), Epstein-Barr virus (EBV), human papillomavirus (HPV), and human T-lymphotropic virus type 1 (HTLV-1) [158]. In recent years, significant progress has been made in mRNA vaccine research targeting HPV. Zhou et al. developed an mRNA-based vaccine against the late oncoproteins E6 and E7 of HPV16, which are abundantly expressed in high-grade squamous intraepithelial lesions (HSIL). In vitro and in vivo studies demonstrated that the translated mRNA was functional and elicited antigen-specific adaptive immune responses. Mice with HPV16 + lesions exhibited tumor growth inhibition, extended lifespan, and the development of protective immune memory following vaccination [159]. Hepatitis B virus (HBV) infection is a major pathogenic factor for liver cancer [160]. Research has shown that mRNA vaccines can activate the innate immune system, inducing the production of potent immunogenicity, high levels of virus-specific antibodies, memory B cells, and T cells, offering prospects for functional cure and prevention of HBV recurrence in chronic patients. However, further in-depth evaluation of mRNA vaccines is needed [161]. HIV infection increases the risk of certain tumors, such as Kaposi's sarcoma and non-Hodgkin's lymphoma [162]. Xie et al. [163] utilized mRNA technology to induce the generation of broadly neutralizing antibody (bnAb) precursors essential for multiple HIV subtypes, providing evidence for the feasibility of germline targeting (GT) and progressive immunization strategies in HIV vaccine development. Through animal models, researchers have successfully elicited specific immune responses to HIV bnAbs, providing new strategies for the development of effective HIV vaccines (Table 5).

**Table 5:** Clinical trials of mRNA vaccines targeting tumors associated viruses

Cancer type		NCT number	Drug administration	Phase	Viruse	Status	Delivery system	Result
Reproductive system tumors	cervical carcinoma	NCT06273553	RG002 Injection (an mRNA Therapeutic Vaccine)	I	HPV16/18	Not yet recruiting	Lipid nanoparticles	Not published
		NCT02116920	mRNA E6/E7	I	HPV genotypes 16, 18, 31, 33 and 45	Not yet recruiting	Lipid nanoparticles	Unknown
		NCT05119855	mRNA-1273 Vaccine	IV	9-valent human papillomavirus (Types 6, 11, 16, 18, 31, 33, 45, 52, 58)	Completed	Lipid nanoparticles	Not published
Skin tumor	Kaposi's sarcoma	NCT05217641	BG505 MD39.3 mRNA, BG505 MD39.3 gp151 mRNA or BG505 MD39.3 gp151 CD4KO mRNA	I	HIV	Active,not recruiting	Lipid nanoparticles	Not published
		NCT02413645	TriMix 100, TriMix 300	I	HIV	Completed	Lipid nanoparticles	The vaccine was secure and well tolerated. There were 31 grade 1/2 and 1 grade 3 adverse events, mostly unrelated to the vaccination. Patients who received the highest dose showed a moderate increase in T-cell responses spanning HTI sequence at week 8. In addition, the proportion of responders receiving any dose of HTI increased from 31% at w0 to 80% postvaccination. The intervention had no impact on caHIV-DNA levels, however, caHIV-RNA expression and usVL were transiently increased at weeks 5 and 6 in the highest dose of iHIVARNA, and these changes were positively correlated with HIV-1-specific-induced immune responses.
		*NCT00833781*	mRNA-transfected autologous dendritic cells	I	HIV	Completed	DC	There were no differences in interferon-gamma enzyme-linked immunospot responses to HIV-1 Gag or Nef in the vaccine or placebo group. CD4 proliferative responses to KLH increased 2.4-fold (*P* = 0.026) and CD8 proliferative responses to KLH increased 2.5-fold (*P* = 0.053) after vaccination. There were increases in CD4 proliferative responses to HIV-1 Gag (2.5-fold vs. baseline, 3.4-fold vs. placebo, *P* = 0.054) and HIV-1 Nef (2.3-fold vs. baseline, 6.3-fold vs. placebo, *P* = 0.009) among vaccine recipients, but these responses were short-lived.
Lymphatic system tumor	Burkitt's lymphoma	NCT05144748	EBV mRNA vaccine	I	EBV	Recruiting	Lipid nanoparticles	Unknown
Digestive System Cancer	Hepatocellular carcinoma	NCT05738447	HBV mRNA vaccine	I	HBV	Recruiting	Lipid nanoparticles	Unknown

### Adjuvants for mRNA vaccines

Adjuvants are additional immunostimulatory agents in vaccines that activate the innate immune system and provide the necessary "help" to increase the magnitude and quality of adaptive responses, thereby offering maximal protection against specific pathogens [164]. Different adjuvants can elicit various immune responses, influencing overall vaccine outcomes. Currently, the adjuvants used in mRNA vaccines generally include four categories: 1) the intrinsic adjuvant effects of mRNA vaccines; 2) mRNAs encoding immunostimulatory molecules; 3) mRNAs encoding antibodies; and 4) adjuvants for mRNA vaccines on the basis of delivery carrier components.

#### The intrinsic adjuvant effect of mRNA vaccines

Exogenous RNA molecules can induce immune responses in mammalian cells. Unmodified exogenous nucleotide mRNAs used to express antigens in mRNA vaccines exhibit intrinsic adjuvant activity by triggering innate immune signalling pathways. Notably, double-stranded RNA (dsRNA) can activate TLR3, while single-stranded RNA is capable of activating mouse TLR7, and RNA oligonucleotides containing thio-phosphorylated nucleotide linkages serve as ligands for human TLR8 [165]. Polyuridine (U) and short dsRNA with 5' triphosphate blunt ends can enhance immune responses through the TLR3 and retinoic acid-inducible gene (RIG)-I signaling pathways without compromising antigen expression, thus functioning as adjuvants for mRNA vaccines [166, 167]. The activation of TLRs and RIG-I signaling can induce the production of proinflammatory cytokines such as tumor necrosis factor-alpha (TNF-α), interleukin-6 (IL-6), IL-12, IL-1β, and interferon-alpha/beta (IFNα/β) (Fig. 1), which enhances the protective immunity required by mRNA vaccines while potentially leading to excessive inflammation [168]. Pioneering work by Kariko et al. demonstrated that unmodified RNA molecules activate TLR or RIG-I signaling pathways, triggering antiviral-like immune responses that may impair RNA translation and promote RNA degradation [15]. Nucleoside-modified mRNA can circumvent this immune activation, such as pseudouridine, which has been widely applied in mRNA vaccines [145, 165]. Recent studies indicate that the modified mRNA in the Pfizer-BioNTech BNT162b2 mRNA vaccine may be recognized by melanoma differentiation-associated protein 5 (MDA-5), triggering IFNα production and contributing to the magnitude of antigen-specific T cell and antibody responses [169].

#### mRNA vaccines encoding immune modulators

Immune modulators typically include cytokines, co-stimulatory molecules, and PRR agonists [170]. Immune modulators such as interferons, interleukins, lymphokines, and tumor necrosis factors play different roles in the immune system. Some trigger inflammation, whereas others support cell growth and differentiation, whereas others enhance lymphocyte functions [171]. It is crucial to restore the anti-tumor immune response by inhibiting immune suppression through the modulation of immune modulators [172]. The use of cytokines in cancer therapy has emerged as a viable treatment option in clinical settings for patients battling cancer [173]. One of the challenges associated with current immunomodulatory treatments is the occurrence of dose-related toxicity resulting from the short half-life of the administered agents, necessitating frequent dosing and systemic distribution, as exemplified by IL-12 therapy [174]. Therefore, intratumoral (IT) and intradermal (ID) injections are often used to induce local immune responses. The transient protein expression and prominent advantages of local delivery make mRNA vaccines complementary to immune modulators, making immune modulators important targets for mRNA vaccines. IL-12, an essential cytokine, can activate CTL and NK cells. In 2018, IL-12 mRNA-LNPs were shown to be effective in hepatocellular carcinoma (HCC) treatment [175]. Due to the unique functions of each cytokine, the efficacy of single cytokine therapy in tumor treatment is limited. Therefore, a combination of multiple cytokines with different functions is often used to improve therapeutic outcomes. Research indicates that mRNA vaccines encoding IL-12 and IL-27 can induce NK and CD8 + T cells within the melanoma tumor microenvironment (TME), demonstrating optimal efficacy [176]. Another study found that a mixture of IL-12, GM-CSF, IL-15, and IFN-α mRNA increased the number of CD4 + T cells and CD8 + T cells in the TME, and adding anti-PD-1 antibody improved mouse survival rates [136]. In 2019, Haabeth et al. [177] pioneered a novel approach to initiate anti-cancer immunity by combining cytokines with co-stimulatory molecules using mRNA. They used a specialized mRNA delivery system to locally express cytokines (CD70, IL-12, and IFN-γ) and co-stimulatory molecules (OX40L, CD80, and CD86) in two tumor models (B-cell lymphoma and colorectal cancer.). Their findings showed that mice given mRNA vaccines containing both cytokines and co-stimulatory molecules achieved complete elimination of tumors, unlike those given other mRNA vaccines that only had partial effects. Combining OX40L with CD80 or CD86, or OX40L with IL-12, notably improved survival rates and delayed tumor growth. These preclinical results indicate that specific cytokines and co-stimulatory molecules could effectively enhance T cell responses against cancer. Currently, most clinical trials on mRNA vaccines encoding immune modulators are in phase I/II to evaluate tolerability. One of the pioneers in this field is eTheRNA, which has developed an adjuvant based on TriMix mRNA consisting of three naked mRNA molecules. Both naked TriMix mRNA evaluated in multiple clinical trials and TriMix mRNA loaded onto DCs ex vivo have shown good tolerability and immunogenicity. Moderna, a leading biotech firm, has created two mRNA therapies enclosed in LNP frameworks to trigger immune responses within tumors. These therapies are undergoing phase I clinical trials to assess the safety and tolerance of repeated administration. One of the products, mRNA-2416, contains mRNA encoding OX40L. It is being tested alone or combined with the intravenous PD-L1 inhibitor durvalumab for treating lymphoma and metastatic ovarian cancer (NCT03323398) [154]. Another candidate, mRNA-2752, comprises OX40L/IL-23/IL-36γmRNA for the treatment of lymphoma (NCT03739931) [178]. Here, OX40L generates secondary signals, enhancing T-cell effector functions and promoting T-cell proliferation and survival. Moderna and AstraZeneca have teamed up to work on the development of MED I1191, which is an IL-12 mRNA product designed for intratumoral administration as part of cancer treatment. Preliminary results from the initial clinical trial revealed that sequential or combination therapy of MED I1191 with durvalumab in patients with advanced solid tumors and skin or subcutaneous lesions is safe and feasible. No treatment-related adverse events leading to treatment discontinuation from MEDI1191 or durvalumab were reported. The combination of MEDI1191 and durvalumab has demonstrated preliminary clinical efficacy; 29.0% of patients achieve either a partial response (PR) or stable disease (SD) for a minimum duration of 12 weeks (NCT03946800) [179] (Table 6).

**Table 6:** Clinical trials of mRNA vaccines encoding immunomodulator

Cancer type		NCT number	Drug administration	Phase	Immunomodulator	Status	Delivery system	Result
Respiratory system tumors (mainly non-small cell lung cancer)	non-small cell lung cancer	NCT02688686	Suppressor of cytokine signaling (SOCS) 1, MUC1 and Survivin mRNA-loaded DC + cytokine-induced killer	I/II	suppressor of cytokine signaling (SOCS) 1	Unknown	DC	No
Skin tumors	Melanoma	NCT01066390	TriMix-DC	I	TLR4, CD40L and CD70	Completed	DC	15 patients had good tolerance, 2 patients had complete remission, and 2 patients had partial remission; It has immunogenicity and long-lasting anti-tumor activity for disease control. Antigen specific CD8 + T cells were detected in 4 out of 5 patients [24]
		NCT00204607	mRNA + GM-CSF	I/II	GM-CSF	Completed	Naked RNA	Not reported
		NCT00204516	mRNA coding for melanoma associated antigens + GM-CSF	I/II	GM-CSF	Completed	Naked mRNA	Not published
		NCT01278940	mRNA-transfected DCs + IL-2	I/II	IL-2	Completed	DC	Not reported
		NCT01530698	autologous dendritic cell vaccine by mRNA Electroporation	I/II	TLR7/8, IL-6	Completed	DC	Not reported
		*NCT04335890*	Vaccination with IKKb matured Dendritic Cells	I	IL-1ß, IL-6 and PGE2	Active,not recruiting	DC	Not reported
		*NCT03394937*	CD40L, CD70, TLR4; tumour-associated antigens: tyrosinase, gp100, MAGE-A3, MAGE-C2, and PRAME	I	CD40L, CD70, TLR4;	Terminated	DC	Good tolerance, low dose (600 μ g) 4/10 and 3/9 of patients with high (1800ug) levels detected vaccine induced immune responses, with immunogenicity in some patients; No adverse reactions of level 3 or above have occurred [32]
		*NCT01676779*	mRNA; b.TAAs: MAGE-A3, MAGE-C2, tyrosinase, gp100	II		Completed	DC	Good tolerance (symptoms: transient local skin reactions, flu like symptoms, shivering after infusion), and may increase the one-year survival rate (71% in the treatment group, 35% in the control group)
		*NCT03291002*	CV8102: TLR7/8, RIG-1	I	TLR7/8, RIG-1	Active,not recruiting	Protamine	Both individual and combined administration showed good therapeutic effects, and local induced immune responses were observed to transform into systemic immune responses
Solid Tumor		*NCT03946800*	MEDI1191 (mRNA encoding IL-12)	I	IL-12	Recruiting	LNP	Preliminary results from the initial clinical trial revealed that sequential or combination therapy of MED I1191 with durvalumab in patients with advanced solid tumors and skin or subcutaneous lesions is safe and feasible. No treatment-related adverse events leading to treatment discontinuation from MEDI1191 or durvalumab were reported. The combination of MEDI1191 and durvalumab has demonstrated preliminary clinical efficacy; 29.0% of patients achieve either a partial response (PR) or stable disease (SD) for a minimum duration of 12 weeks [179]
		*NCT04455620*	BNT151 (mRNA encoding IL-2)	I/II	IL-2	Recruiting	LPX	Ongoing
		*NCT04710043*	BNT152 (mRNA encoding IL-7) plus BNT153 (mRNA encoding IL-2)	I	IL-7/IL-2	Recruiting	LPX	Ongoing
		*NCT05392699*	ABOD2011 (mRNA encoding IL-12)	I	IL-12	Recruiting	Naked-mRNA	Ongoing
Neurological tumors(mainly glioblastoma)	glioblastoma	NCT03396575	TTRNA-DC vaccines with GM-CSF	I	GM-CSF	Recruiting	DC	No
		NCT02465268	HCMV pp65-shLAMP or pp65-flLAMP + temozolomide, GM-CSF, and Td	II	GM-CSF	Recruiting	DC	Not published
		NCT04963413	Autologous DCs derived from PBMC loaded with RNA encoding the human CMV matrix protein pp65-LAMP plus GM-CSF	I	GM-CSF	Active,not recruiting	DC	Not published
		NCT00626483	CMV pp65-LAMP mRNA-loaded DC + GM-CSF	I	CMV pp65-LAMP	Completed	DC	Not published
		NCT03927222	Human CMV pp65-LAMP mRNA-pulsed autologous DCs + temozolomide + tetanusdiphtheria toxoid + GM-CSF	II	CMV pp65-LAMP	Recruiting	DC	Not published
Urinarysystem tumor (mainly prostatecancer)	Prostate cancer	NCT02452307	Peptide vaccine + montanide ISA-51 + / − GM-CSF + / − imiquimod + / − mRNA/protamin	I/II	GM-CSF	Unknown	Protamine	No
Blood System Cancer(leukemia mainly)	leukemia	NCT00514189	Autologous dendritic cells	I	GM-CSF	Terminated	DC	No
		NCT02693236	Adenovirus-transfected autologous DCs + CIK cells	I/II	cytokine-induced killer (CIK) cell	Unknown	DC	No
Digestive System Cancer	Colorectal Cancer with Liver Metastases	NCT04157127	Pancreatic adenocarcinoma mRNA and lysate With standard therapy	I	Th-1	Recruiting	DC	No
		*NCT03323398*	mRNA-2416:OX40L	I/II	OX40L	Active, not recruiting	Lipid nanoparticles	Good safety and tolerability, with no occurrence of > Level 3 adverse reactions; 14/39 patients were in stable condition, and 4/6 patients with ovarian cancer were in stable condition. The patients receiving treatment showed that OX40L protein and T cell infiltration in the tumor microenvironment increased, PD-L1 transcription was up-regulated, and the expression of proinflammatory genes was activated [154].
	Colon Cancer Gastrointestinal Cancer	NCT03739931	mRNA-2752:OX40L, IL-23, IL-36Y	I	OX40L, IL-23, IL-36Y	Recruiting	Lipid nanoparticles	Good tolerance; Tumor shrinkage is related to drug use. 0.5 mg RNA combined with Duvalimab, 81% of bladder cancer focus regression was observed; Treatment has a sustained immune regulatory effect, with elevated levels of IFN-y, TNF-a, and PD-L1 detected in tumors and plasma [178].
Other Cancer	Ductal carcinoma in situ	NCT02872025	mRNA-2752 (mRNA encoding OX40L, IL-23, and IL-36γ)	I	OX40L, IL-23, and IL-36γ	Recruiting	LNP	well tolerated with slight dose-limiting toxicities

#### mRNA vaccines encoding antibodies

Since the development of monoclonal antibodies (mAb) using hybridoma technology in 1975, antibodies have risen to prominence as a rapidly expanding category of pharmaceuticals that specifically target cancer cells [180]. These antibodies have anti-tumor effects through mechanisms such as antibody-dependent cell-mediated cytotoxicity (ADCC), antibody-dependent cellular phagocytosis (ADCP), complement-dependent cytotoxicity (CDC), and blockade of immunosuppressive signals. Conventional antibodies consist of Fab and Fc fragments, with Fab binding to tumor antigens and the Fc region interacting with FcγR on NK cells and macrophages to facilitate cancer cell lysis. In addition to traditional antibodies, single-chain variable fragments (scFvs), single-domain antibodies (sdAbs), and bispecific antibodies (bsAbs) have demonstrated potential in immunotherapy. BsAbs can form T-cell–bsAb–tumor cell complexes, mediating immune cell-mediated killing [181]. Researchers at CureVac studied mRNA vaccines targeting antibodies. After 9 years, they were able to develop mRNA vaccines targeting antibodies that effectively reduced tumor growth in a mouse lymphoma model, supporting the use of mAb-targeting mRNA vaccines in cancer immunotherapy [182]. Leiba-Kasper and colleagues conducted a study to explore the intricate relationship between the absorption, distribution, metabolism, and excretion of the mRNA-encoded anti-HER2 antibody trastuzumab, elucidating its impact on the body and its ability to combat cancer. Through their research, they confirmed the potent anticancer properties of this novel therapeutic approach, shedding light on the mechanisms underlying its efficacy in targeting HER2-positive tumors. The findings from this investigation serve to validate the promising therapeutic potential of mRNA-encoded antibodies in the fight against cancer, opening new avenues for optimized treatment strategies and improved patient outcomes [183]. In addition to monoclonal antibodies, a range of mRNA-encoded bispecific antibodies (bsAbs) have been developed. CCL2 and CCL5 play critical roles in tumor-associated macrophage (TAM) accumulation and HCC immunosuppression. The Wang group developed the bispecific antibody BisCCL2/5i, which targets CCL2 and CCL5, promoting TAM polarization towards the anti-tumor M1 phenotype and reversing immune suppression in the tumor microenvironment (TME). BisCCL2/5i sensitizes HCC to PD-L1 blockade and prolongs survival in a murine model of liver malignancy [184].Bi-specific T cell engagers (BiTEs) are a class of bispecific antibodies lacking an Fc region, consisting of two scFv domains—one recognizing CD3 and the other binding to the cancer cell target antigen—facilitating T cell-mediated tumor killing [185]. The Staid team has developed the RiboMab platform, which includes BiTE mRNA targeting three tumor-associated antigens (TAA)—CD3 × CLDN6, CLDN18.2 × CD3, and EpCAM × CD3. The mRNA encoding CD3 × CLDN6 BiTE exhibits a longer half-life in serum compared to the protein counterpart, leading to complete tumor regression in a mouse model without eliciting systemic immune reactions [186]. CD3 × CLDN6 mRNA (BNT142) is currently undergoing Phase I/II clinical trials (NCT05262530) [179]. While research on mRNA vaccine-encoded antibodies remains limited, monoclonal antibodies (mAbs) and bispecific antibodies (bsAbs) have already shown efficacy. By encoding anticancer antigens, blocking immune checkpoint molecules, and mediating T-cell anti-tumor responses through mRNA vaccine-encoded antibodies, the potential of mRNA antibody immunotherapy is vast. (Table 7).

**Table 7:** Clinical trials of mRNA vaccines encoding Ab

Cancer type		NCT number	Drug administration	Phase	Status	Delivery system	Result
Solid Tumor		NCT05262530	BNT142 (mRNA encoding antibodies targetingCD3 × CLDN6)	I	Recruiting	LNP	Ongoing
Reproductive system tumors (mainly ovarian cancer)	ovarian cancer	NCT04683939	BNT141 (mRNA encodinganti-Claudin18.2 monoclonal antibody)	I/II	Recruiting	LNP	Ongoing

#### Adjuvants for mRNA vaccines based on delivery carrier components

Cationic lipids may play a critical role in the adjuvant activity of lipid nanoparticles (LNPs). LNPs based on the ionizable cationic lipid DLinDMA exhibit immunostimulatory properties and serve as adjuvants for nucleoside-modified mRNA vaccines, effectively eliciting follicular helper T (TFH) cell responses and germinal centre B-cell responses that produce neutralizing antibodies [187]. The cationic lipid-like substance C1 facilitates the delivery of mRNA into cells, promoting the release of inflammatory cytokines such as IL-1β, IL-6, and IL-12P70 and upregulating the expression of costimulatory molecules via the TLR4 signalling pathway [141]. Lipid C12-TLRa, containing a TLR7/8 agonist, enhances mRNA vaccine delivery and TLR responses, collectively inducing high levels of neutralizing antibodies [188]. Another ionizable lipid-like substance, A2-Iso5–2DC18 (A2), activates STING signaling and releases cytokines such as CXCL10, thereby enhancing the immune response [68]. The non-nucleotide STING agonist-derived amino lipid SAL12, formulated into LNPs, induces the production of IFNβ, triggering potent neutralizing antibodies against SARS-Cov-2 [189]. Additionally, the direct incorporation of all-trans retinoic acid (ATRA) during LNP self-assembly results in ATRA-LNPs that effectively activate dendritic cells, eliciting robust systemic T-cell responses and increasing the infiltration of antigen-specific cytotoxic T cells in colorectal tumors [190]. However, the intrinsic immunostimulatory properties of lipid materials are not always beneficial for vaccines. A study reported that lipid components (DOTMA and DOPE) in mRNA vaccines promote mitochondrial ROS production in monocytes, activating the NLRP3 inflammasome and releasing IL-1β, leading to inflammatory side effects [191]. These findings underscore the importance of selecting lipid components with appropriate immunostimulatory effects for the rational design and development of future mRNA vaccines.

### Combined application of mRNA vaccines with other tumor targeted therapies

Currently, many patients have developed resistance to tumor-targeted monotherapies, substantially impacting the effectiveness of tumor-targeted therapy. Therefore, the combined application of mRNA vaccines with other tumor-targeted therapies holds tremendous potential in enhancing treatment outcomes. By harnessing the synergistic effects of different therapeutic approaches, this combination strategy offers a promising avenue for overcoming resistance and improving the overall efficacy of tumor-targeted therapy.

#### Combined application of mRNA Vaccines and Adoptive Cell Therapy (ACT)

ACT involves extracting immune-active cells from cancer patients, culturing and evaluating their function outside the body, and finally reintroducing them back into patients to target and destroy tumors directly [192]. Adoptive immune cell therapy mainly includes several categories such as TCR-T and CAR-T [193]. Currently, the most commonly used/most effective applications are CAR-T therapy and TCR-T therapy [194, 195].TCR-T cell therapy entails the isolation of T cells from the patient's body, genetic engineering to express a specific T-cell receptor (TCR), and targeting tumor-associated antigens for recognition and elimination [196]. mRNA vaccines have the ability to induce a broad immune response, encompassing humoral and cellular immunity, while TCR-T therapy allows for direct targeting and destruction of tumor cells [197]. If these two therapies are utilized in conjunction, it has the potential to enhance the anti-tumor capabilities of the innate immune system and directly target [198] specific tumor antigens, thereby demonstrating synergistic efficacy. Furthermore, as a relatively safe and repeatable administration mode, mRNA vaccines could enhance the tolerability of TCR-T cell therapy [199]. However, the joint application of mRNA vaccines and TCR-T cell therapy is still in its early exploratory phase [200].CAR-T is a novel immunotherapy approach that employs genetic engineering technology to modify T cells, allowing them to exert anti-tumor effects [201]. Specifically, CAR-T cells constitute a cutting-edge immunocellular therapy that uses genetic engineering to insert customized chimeric antigen receptors (CARs) into T cells. CARs are generated by combining exogenous antigen recognition domains with T-cell receptor domains. This fusion enables CAR-T cells to accurately target and destroy specific cancer cells. Clinical trials have demonstrated promising outcomes with CAR-T-cell therapy, providing new treatment options for cancer patients. These CAR structures consist of single-chain antibody extracellular domains, extracellular hinge domains, transmembrane domains, and intracellular domains, which facilitate their recognition and binding of specific antigens. CAR-T-cell therapy involves transfecting CAR-T cells with mRNAs encoding target proteins to produce the mRNA‒target-CAR-T complex, which is subsequently administered to the body. This therapeutic strategy has been investigated in cancer patients, and promising results have been reported. For instance, a study by Tchou et al. [202] demonstrated that T cells transfected with CAR mRNA targeting c-Met exhibited good tolerance within the breast tumor tissues and were capable of triggering an inflammatory response. This finding suggested that the combination of CAR-T-cell therapy and mRNA vaccines could be a viable treatment approach, enabling more comprehensive and precise targeting of tumors for greater tumor killing efficacy. In another study, Beatty et al. [203]evaluated T cells transfected with mRNA encoding mesothelin-directed CAR as a potential treatment for pancreatic tumors. Interestingly, in a phase 1 study, these cells did not cause CRS or trigger neurological symptoms. Previous studies have indicated that IVT mRNAs encoding TAMs can be directly delivered into tumors via CAR-T nanoparticle technology to induce local regulation of tumor-associated dendritic cells (TADCs) [204].

#### mRNA vaccines combined with Immune Checkpoint Inhibitor (ICI)

Immune checkpoints play crucial protective roles in regulating the human immune system, acting as brakes to prevent excessive T-cell activation and other undesirable effects. However, tumor cells frequently exploit this regulatory mechanism by overexpressing immune checkpoint molecules, which effectively dampen immune system responses, evade immunosurveillance, and promote tumor development. The most widely researched and applied ICIs include CTLA4, PD-1, and PD-L1. ICI therapy works by blocking immune checkpoint activity and activating T cells to attack tumors, leading to anti-tumor effects. Furthermore, ICIs can maintain induced immunological responses while inhibiting the induction of T-cell depletion indicators, making them useful partners for mRNA vaccines [205]. Ugur Sahin et al. [206] demonstrated that combining the melanoma mRNA vaccine FixVac with PD-1 inhibitors can result in a synergistic effect. Surprisingly, drug sensitivity can even be restored in patients who have previously developed resistance to ICI treatment using this combination treatment. This trial involved 89 advanced melanoma patients (phase IV) who were treated with at least one vaccine targeting a TAA and who had received one or more ICI therapies. All patients received 8 FixVac vaccinations. Interestingly, among them, 47 out of 89 patients (52.81%) displayed positive responses, with 42 exhibiting the best objective response and 5 exhibiting partial reactions. In addition, 3 patients achieved partial remission, 7 patients remained stable, and 1 patient achieved complete remission of the metastatic lesion among the 25 patients who received FixVac monotherapy. Moreover, among the 17 patients treated with FixVac and PD-1 inhibitors, 6 experienced partial reactions and target lesion regression at all doses. During the two-year follow-up, the majority of patients who achieved partial remission or remained stable had longer disease control. Furthermore, in another important clinical trial, researchers used the mRNA-4157/V940 vaccine in combination with pembrolizumab. The results revealed a significant decrease in the risk of disease relapse among patients who were administered combination therapy compared with those who were solely treated with PD-1 inhibitors [207]. In another study, Lina Liu et al. [208] reported that MUC1-based mRNA vaccination can successfully activate CTL responses against triple-negative breast cancer (TNBC). Furthermore, combining an mRNA vaccine with an anti-CTLA-4 monoclonal antibody can markedly enhance the T-cell immune response, and the effect was substantially superior to that of treatment with an mRNA vaccine alone or anti-CTLA-4 monoclonal antibody therapy alone. Although research on these technologies is still relatively limited, their prospects are highly promising. Furthermore, small interfering RNA (siRNA) has shown great potential in ICIs [209], which can be encapsulated in the same vector as mRNAs to prevent repeated delivery, have shown great potential in the treatment of ICIs. Although there are several limitations associated with its small molecular weight and low encapsulation efficiency, the encapsulation concentration of this therapy is still within acceptable limits.

#### Combination application of mRNA vaccines and oncogene therapy

Oncogene therapy often involves introducing wild-type copies of tumor suppressor genes or exploiting tumor-specific phenotypic changes to selectively target cancer cells. Tumor suppressor genes (TSGs) are essential for maintaining genomic integrity and regulating cell growth, differentiation, and apoptosis [210]. The loss of TSG function is commonly associated with the occurrence, progression, and treatment resistance of cancer [211]. Furthermore, numerous cancer driver genes, mostly TSGs, have been identified through human cancer exon sequencing studies [212]. The majority of TSGs experience functional loss, leading to overactivation of cancer phenotypes through the aforementioned pathways. In such scenarios, a potential therapeutic approach involves suppressing downstream pathways via supplementation with TSGs. However, difficulties in delivery, genomic integration, and mutation risks pose significant obstacles to gene therapy when functional copies are restored via DNA transfection. mRNA vaccines have been demonstrated to effectively address these issues. In a study from 2018, a PTEN-mRNA vaccine was encapsulated in polyethylene glycol (PEG)-coated polymer‒lipid hybrid nanoparticles (LNPs), successfully introducing PTEN-deficient prostate cancer cells. The therapeutic efficacy of inhibiting the PI3K/Akt signalling pathway and promoting cancer cell apoptosis has been validated [213]. In a study conducted in 2021, PTEN mRNA-NPs were shown to restore the protein expression and autophagy of PTEN-deficient cancer cells, demonstrating therapeutic effects against melanoma and PD-1-resistant prostate cancer [214]. While the utilization of TSG-mRNA vaccines remains largely uncharted territory, their efficacy has been demonstrated in various mouse cancer models, underscoring their considerable practical promise.

## Future development trends of mRNA vaccines in tumor targeted therapy

mRNA vaccines, as an emerging immunotherapeutic modality, exhibit a diversified and promising outlook in their future development trends [215]. Here, we discussed the importance and impact of personalized vaccine design, multifunctionality, combination therapy strategies, mucosal immunity, and nanotechnology on the application of mRNA vaccines in targeted tumor therapy. Personalized mRNA tumor vaccines design stands out as a research hotspot [143]. By elucidating the genetic and immune characteristics of patient tumor cells, highly personalized mRNA tumor vaccines can be tailored for individual patients. These customized vaccines can more precisely trigger patient-specific immune responses, thereby enhancing treatment efficacy. In the future, interdisciplinary studies encompassing genomics, immunomics, and bioinformatics will provide a more precise theoretical foundation for personalized vaccine design, laying solid groundwork for clinical applications. The development of personalized vaccines will also benefit from the continuous advancement of high-throughput sequencing technologies, which will aid in the rapid and accurate detection of genomic information in individual tumors. Furthermore, the application of artificial intelligence technology will provide more support. For example, optimizing mRNA sequences via artificial intelligence technology has greatly reduced the immunogenicity of mRNA vaccines [216]. By integrating research findings from different interdisciplinary fields, the design of personalized vaccines will continue to be optimized, providing more effective treatment strategies for a vast number of cancer patients. In addition to directly activating the immune system to attack tumors, the future development trend of mRNA vaccines also includes achieving vaccine multifunctionality. These findings indicate that mRNA vaccines can also regulate the immune microenvironment, inhibit tumor growth and spread, and perform other functions. By incorporating various active components, such as immune modulators and cytokines, mRNA-based tumor vaccines will gradually achieve comprehensive intervention against tumors, suggesting new possibilities for cancer treatment [153]. Future research will focus on deciphering the interaction mechanisms of different components in vaccines to achieve more precise and efficient therapeutic effects. Simultaneously, through techniques such as gene editing, the active components in vaccines may undergo more precise regulation, further enhancing the multifunctional effects of the vaccines. Research on multifunctional vaccines will provide broader insights for the development of personalized treatment strategies. In the context of mRNA-based tumor vaccines, the integration of multiple therapeutic modalities is foreseen to emerge as a pivotal and compelling avenue for improvement [217]. The combination of mRNA vaccines with other anti-tumor treatment modalities holds the promise of further enhancing the therapeutic efficacy of vaccines and achieving synergistic effects via multiple treatment mechanisms. In the future, interdisciplinary research teams will conduct more basic research and clinical trials to explore the mechanisms and application prospects of combined therapeutic strategies in tumor treatment. Interdisciplinary collaboration has become a key approach in the study of combined therapeutic strategies, involving experts from multiple disciplines, including immunology, cell biology, pharmacology, and others. Research on combined therapeutic strategies not only requires a deep understanding of the mechanisms of different treatment modalities but also aims to explore how to rationally combine these modalities to achieve optimal effects in the treatment of tumors at different stages. Mucosal immunology, as a new direction in mRNA tumor vaccine research, has garnered significant attention [218]. By guiding immune responses in local mucosal tissues and designing specific mucosal antigens and adjuvants, prevention and treatment of tumors can be achieved. The introduction of mucosal immunization strategies will lead to novel ideas and possibilities in the field of tumor prevention and control, providing broader insights for the research and application of future tumor vaccines. In future research on mucosal immunology, a deeper exploration of the characteristics of mucosal immune tissues and their relevance to tumor-targeted therapy will be carried out. Additionally, customized mucosal immune vaccines are crucial for enhancing the delivery efficiency and immune effects of vaccines in mucosal immune tissues. Furthermore, interdisciplinary collaborations will open new avenues for the application of mucosal immunization in tumor vaccines. The application of nanotechnology in mRNA tumor vaccines has also attracted considerable attention. Nanocarriers can safely and effectively deliver mRNA vaccines into the body, increasing their bioavailability and immunogenicity [219]. Concurrently, the targeted drug delivery and reduced side effects of nanotechnology significantly increase the therapeutic efficacy and safety of tumor vaccines. The combination of nanotechnology and mRNA-based tumor vaccines will lead to the development of novel therapeutic strategies and possibilities for cancer treatment. Future developments in nanotechnology will focus on improving the stability and targeting of carriers, further reducing their metabolism and excretion rates in the body, thereby prolonging the vaccine's efficacy and impact. Additionally, nanotechnology can provide more possibilities for the modification and functionalization of vaccines to meet the diverse treatment needs of different tumor types and individual patients. The application of nanotechnology in mRNA tumour vaccines will introduce more precise and efficient therapeutic approaches to the field of cancer treatment. Despite the significant potential of mRNA vaccines in targeted cancer therapy, several limitations persist: 1) Poor stability: the chemical structure and biological properties of mRNAs render them inherently unstable and susceptible to degradation, which affects their biological activity and immunogenicity [220]. Although chemical modifications can increase stability, their efficacy is limited, with studies indicating that N1-methylpseudouridine-modified mRNAs undergo ribosomal frameshifting during translation [221]. 2) Low in vivo delivery efficiency: The safe and effective delivery of mRNA to target cells is crucial for its functionality [222]. Current delivery systems, such as liposomes and lipid nanoparticles, have improved in stability and intracellular delivery [67], yet the efficiency of delivery remains suboptimal [152]. 3) Complex tumor immune evasion mechanisms: Tumor cells employ multiple strategies to evade immune surveillance [223], presenting a challenge in the field of mRNA vaccine-based targeted cancer therapy [24]. 4) High interindividual variability: Differences in genetic background, immune status, and disease conditions among individuals influence the immunogenicity and therapeutic efficacy of mRNA vaccines [224]. Based on the aforementioned limitations, we advocate for the following enhancements to mRNA vaccines: Firstly, the essence of mRNA vaccines lies in harnessing the endogenous cellular machinery for antigen protein synthesis to trigger immune responses. The optimization of mRNA structure and sequence can significantly enhance its stability and transcriptional efficiency [216, 225]. Researchers have improved mRNA expression levels and duration within cells by refining the 5' cap structure, 3' poly(A) tail, codon usage, and nucleotide modifications [154, 215, 226]. Secondly, beyond optimizing the mRNA itself, the adoption of novel delivery systems is pivotal for enhancing the immunogenic efficacy of mRNA vaccines. Consequently, the development of safer and more effective new carriers is imperative. Emerging lipid nanomaterials, such as biodegradable fatty acid nanoparticles, have demonstrated superior targeting and immunogenicity in animal models [227]. Similarly, as previously noted, biomimetic carriers can enhance the efficiency and intensity of vaccine mRNA translation compared to conventional materials [113].Finally, for mRNA to be translated into antigen proteins, it must successfully traverse into the cytoplasm of target cells, a process fraught with challenges such as lysosomal degradation post-endocytosis or nuclease degradation [228].Therefore, enhancing mRNA stability and penetrance is crucial [229]. For example, physical methods like electroporation can temporarily disrupt the cell membrane, thereby facilitating increased mRNA transport efficiency [230]. We also discuss key points to consider in the clinical translation of mRNA vaccine technology. First, there is a critical need to strengthen preclinical research to thoroughly investigate the biological characteristics of the vaccine, including its stability, immunogenicity, and routes of administration, to ensure its safety and efficacy. Second, conducting large-scale randomized controlled trials is essential for validating the differences between mRNA vaccines and traditional vaccines, particularly in terms of administration routes, dosage design, and immunogenicity. Furthermore, addressing immune responses across different populations can provide scientific evidence for the precise use of vaccines, ensuring their preventive efficacy and safety. Third, the assessment of long-term efficacy and safety is vital. Continuous monitoring of the duration of immunity and potential adverse reactions postvaccination, especially rare severe events, is necessary to obtain long-term immunogenicity, protective efficacy, and safety data through systematic follow-up analysis, thus providing a reliable basis for clinical applications. Finally, optimizing the design of clinical trial protocols is essential to increase the scientific rigor and reliability of trials. The administration routes and treatment regimens should be flexibly designed according to the characteristics of the vaccine, and trial standards and observational indicators should be optimized on the basis of the immunological characteristics of different populations, with endpoint indicators determined in conjunction with epidemiological considerations.

## Conclusion

mRNA vaccines represent a promising solution to overcome the limitations encountered in conventional cancer immunotherapy, offering enhanced and durable treatment alternatives. We posit that the integration of mRNA vaccine technology into tumor-targeted therapy will yield expanded applications, serving as an effective tool in the battle against cancer. The versatility of mRNA vaccines, coupled with their ability to elicit immune responses targeting specific tumor antigens, holds great potential for personalized cancer treatment strategies. As research continues to advance in this area, it is conceivable that mRNA vaccines will play a pivotal role in tumor-targeted therapy.

## Data Availability

Not applicable.

## Abbreviations

mRNA: Messenger ribonucleic acid
ICI: Immune checkpoint inhibitors
SAM: Self-amplifying
ORF: Open reading frame
NSP: Non-structural protein
UTR: Untranslated region
IVT: In vitro transcription
TLR: Toll like receptors
dsRNA: Double-stranded RNA
HPLC: High performance liquid chromatography
FPLC: Fast protein liquid chromatography
ARCA: Anti-Reverse Cap Analog
DC: Dendritic cells
CNE: Cationic nanoemulsion
CPP: Cationic cell penetrating peptides
LNP: Lipid nanoparticle
LP: Liposomes
DSPC: Double stearyl phosphatidylcholine
PEI: Polyethylene imine
PAMAM: Polyamide amine
PPI: Pol (propylene imine)
PAE: Poly (amino ester)
PEG: Polyethylene glycol
PLGA: Poly(lactic-co-glycolic acid)
PTEN: Phosphatase and tensin homologue
PLA: Polylatic acid
PBAE: Poly β-aminoesters
PGLA: POLY(D,L-LACTIDE-CO-GLYCOLIDE)
VRP: Virus like replicon particle
DOTAP: 1.2-Diol sn glycerol-3-phosphate choline
VEEV: Venezuelan equine encephalitis virus
MHC: Main Histocompatibility complex
GMCSF: Granulocyte macrophage colony-stimulating factor
LPX: Lipid complex
TAA: Tumor associated antigens
APC: Antigen presenting cell
HLA: Human leukocyte antigen
BMDC: Bone marrow-derived dendritic cell
OVA: Ovalbumin
PRR: Pattern recognition receptor
PAMP: Pathogen associated molecular pattern
ssRNA: Single strand RNA
TCR: T cell receptor
RIG-I: Cytosolic retinoic acid inducible gene I
RLR: Cytosolic retinoic acid inducible gene I (RIG-I) like receptor
MDA 5: Melanoma differentiation associated gene 5
IFN: Interferon
CA: Carbohydrate antigen
AFP: Alpha fetoprotein
CAR-T: Chimeric Antigen Receptor T-Cell Immunotherapy
TADC: Tumor associated dendritic cell
CTLA: Cytotoxic T-lymphocyte-associated protein
PD-1: Programmed cell death protein 1
SiRNA: Small interfering RNA
TME: Tumor microenvironment
MDSC: Myeloid suppressor cell
CAF: Cancer associated fibroblast
NK cell: Natural killer cell
TIPE2: Tumor necrosis factor- α Induced protein 8-like 2
TIME: Tumor immune microenvironment
TNBC: Triple negative breast cancer
CTLs: Cytotoxic T lymphocyte
TNF: Tumor necrosis factor
IL: Interleukin
TFH: Follicular helper T
BiTEs: Bi-specific T cell engagers
scFvs: Single-chain variable fragments
sdAbs: Single-domain antibodies
bsAbs: Bispecific antibodies
ADCP: Antibody-dependent cellular phagocytosis
CDC: Complement-dependent cytotoxicity
mAb: Monoclonal antibodies
bnAb: Broadly neutralizing antibody
HSIL: High-grade squamous intraepithelial lesions
HBV: Hepatitis B virus
HCV: Hepatitis C virus
EBV: Epstein-Barr virus
HPV: Human papillomavirus
HTLV-1: Human T-lymphotropic virus type 1
GMCSF: Granulocyte–macrophage colony-stimulating factor
EGFR: Epidermal growth factor receptor
hTERT: Human telomerase reverse transcriptase
NY-ESO-1: New York Esophageal Squamous Cell Carcinoma-1
MAGE: Melanoma-associated antigen
LC: Langerhans cell
HLA: Human leucocyte antigen
PAP: Prostatic Acid Phosphatase
PSA: Prostate specific antigen
KLH: Keyhole Limpet Hemocyanin
PSCA: Prostate Stem Cell Antigen
PSMA: Prostate Specific Membrane Antigen
STEAP: Six-segment transmembrane epithelial antigen of prostate
WT: Williams Tumor
PRAME: Preferentially expressed antigen in melanoma
CMV: Cytomegalovirus
LAMP1: Lysosomal Associated Membrane Protein 1
IDO: Indoleamine 2,3-dioxygenase
KRAS: V-Ki-ras2 Kirsten ratsarcoma viral oncogene homolog
SOCS: Suppressor of cytokine signaling
CD40L: CD40 Ligand
PGE2: Prostaglandin E2
CIK: Cytokine-induced killer cells
OX40L: TNF Receptor Superfamily Member 4 Ligand
CLDN6: Claudin-6

## Data availability

No datasets were generated or analysed during the current study.
